# Structure, expression differentiation and evolution of duplicated fiber developmental genes in *Gossypium barbadense *and *G. hirsutum*

**DOI:** 10.1186/1471-2229-11-40

**Published:** 2011-02-25

**Authors:** Huayu Zhu, Xiaoyong Han, Junhong Lv, Liang Zhao, Xiaoyang Xu, Tianzhen Zhang, Wangzhen Guo

**Affiliations:** 1 National Key Laboratory of Crop Genetics & Germplasm Enhancement, Cotton Research Institute, Nanjing Agricultural University, Nanjing 210095, China

## Abstract

**Background:**

Both *Gossypium hirsutum *and *G. barbadense *probably originated from a common ancestor, but they have very different agronomic and fiber quality characters. Here we selected 17 fiber development-related genes to study their structures, tree topologies, chromosomal location and expression patterns to better understand the interspecific divergence of fiber development genes in the two cultivated tetraploid species.

**Results:**

The sequence and structure of 70.59% genes were conserved with the same exon length and numbers in different species, while 29.41% genes showed diversity. There were 15 genes showing independent evolution between the A- and D-subgenomes after polyploid formation, while two evolved via different degrees of colonization. Chromosomal location showed that 22 duplicate genes were located in which at least one fiber quality QTL was detected. The molecular evolutionary rates suggested that the D-subgenome of the allotetraploid underwent rapid evolutionary differentiation, and selection had acted at the tetraploid level. Expression profiles at fiber initiation and early elongation showed that the transcripts levels of most genes were higher in Hai7124 than in TM-1. During the primary-secondary transition period, expression of most genes peaked earlier in TM-1 than in Hai7124. Homeolog expression profile showed that A-subgenome, or the combination of A- and D-subgenomes, played critical roles in fiber quality divergence of *G. hirsutum *and *G. barbadense*. However, the expression of D-subgenome alone also played an important role.

**Conclusion:**

Integrating analysis of the structure and expression to fiber development genes, suggests selective breeding for certain desirable fiber qualities played an important role in divergence of *G. hirsutum *and *G. barbadense*.

## Background

Cotton (*Gossypium *spp.) is the world's most important fiber crop plant. While most of the > 50 *Gossypium *species are diploid (n = 13), five are allopolyploids (n = 26), originating from an interspecific hybridization event between A- and D-genome diploid species. Humans have independently domesticated four different species for their fiber, two of which are diploids, *Gossypium herbaceum *and *G. arboreum*, and two are allopolyploids, *G. hirsutum *and *G. barbadense *[1].

Alhough *G. hirsutum *and *G. barbadense *probably originated from a single hybridization event between A- and D- diploid species, the two have very different agronomic and fiber quality characteristics. The high yield potential and diverse environmental and production system adaptability of *G. hirsutum *make it the most widely cultivated species, accounting for about 97% of the world's cotton fiber [2]. *G. barbadense *is a more modern species possessing superior fiber quality. Novel alleles are responsible for the improved fiber quality in *G. barbadense*. Despite its higher fiber quality, however, the narrow adaptation range and low yield of *G. barbadense *limit its cultivation. The two *Gossypium *species are sexually compatible, although partial sterility, longer maturity, and hybrid breakdown are often observed in later generations [3]. Nonetheless, the introgression of favorable alleles from *G. barbadense *to *G. hirsutum *would likely improve the fiber quality of *G. hirsutum *while simultaneously maintaining its high fiber yield [4].

The cotton fiber is a single cell without the complex cell division and multicellular development that develops from ovule's epidermal cells. Fiber development occurs in four distinct, but overlapping stages: initiation, elongation, secondary wall synthesis, and maturation [5]. To date, many of the genes predominantly expressed in cotton fiber development have been isolated and characterized. *Gh14-3-3L *was found to be predominantly expressed during early fiber development, and may be involved in regulating fiber elongation [6]. Yoder et al. [7] defined pectate lyase (PEL) as a cell wall modification enzyme. *GhPel *was found to play an essential role in fiber cell elongation by degradation of the de-esterified pectin for cell wall loosening [8]. Ruan et al. [9] suggested the sucrose synthase gene (*Sus*) played an important role in the initiation and elongation of single-celled fibers by inﬂuencing carbon partitioning to cellulose synthesis. *GhBG *(β-1,4-glucosidase), one of three cellulases, was specifically expressed in fiber cells and plays an important role in degradation of the primary cell wall and promotion of secondary cell wall synthesis [10]. Cotton *CelA1 *and *CelA2 *genes, encoding the catalytic subunit of cellulose synthase, are expressed at high levels during active secondary wall cellulose synthesis in developing cotton fibers [11]. Two cotton *Rac *genes, *GhRacA *and *GhRacB*, expressed in the fibers at the initiation and elongation stages, might play an important role in early fiber development [12]. In addition, several genes are expressed specifically or preferentially in fibers [13-18], although their exact functional roles remain unclear.

In theory, there are two homologs in tetraploid cotton species, representing descendants from the A-genome and D-genome donors at the time of polyploidy formation. The goals of this study were to: 1) better understand the genetic basis of cotton fiber development, 2) identify the structural difference of duplicated genes, and 3) reveal the expression and evolution of fiber quality differences between upland and sea-island cotton. To complete this study, we selected 17 fiber development genes accessioned in National Center for Biotechnology Information (NCBI, http://www.ncbi.nlm.nih.gov) to study structure and expression differences of the two cultivated tetraploid species. To investigate their frame and sequence divergence, we initially cloned these genes in the genome DNA of the *G. hirsutum *accession TM-1, the *G. barbadense *cultivar Hai7124, and their two putative diploid progenitors. The chromosomal locations of each homeolog of several studied genes, having effective single nucleotide polymorphism (SNP) or amplification polymorphism loci between TM-1 and Hai7124 were determined by linkage analysis in allotetraploid cotton using an interspecific BC_1 _mapping population (TM-1×Hai7124)×TM-1] [19-22]. Finally, expression patterns of each gene and each homeolog were explored. A more thorough understanding of interspecific divergence of cotton will provide a solid foundation from which key fiber quality genes may be exploited in cotton molecular breeding.

## Results

### Sequence and structure analysis of fiber development genes

The orthologs of each of the 17 genes were cloned and sequenced (GenBank accession numbers (GQ340731-GQ340736, HQ142989-HQ143048, and HQ143055-HQ143090; Table 1). Phylogenetic groupings and sequence comparisons allowed the copy number for all genes, except *Exp1 *in the Hai7124 cultivar, to be independently isolated with a single copy from the diploid species and two homeologs from each tetraploid species for each gene. There was a single copy of *Exp1 *in the diploid species and two distinct copies in TM-1, however, the *Exp1 *sequence from Hai7124 was of only one type, though more than 10 clones were selected randomly to sequence. This result was further validated by a different primer pair of this gene (see Additional file 1: Supplemental Table S1 for list of primer pairs). The sequence from Hai7124 has a closer relationship with *G. raimondii *than with *G. herbaceum*. Further, southern blotting of *Exp1 *performed on the four species, showed two distinct hybridizing bands after digestion with *Eco*RI and *Hind*I in Hai7124 and TM-1, and one hybridizing band in *G. herbaceum *and *G. raimondii *(Figure not shown), which indicated that *Exp1 *had two copies in both TM-1 and Hai7124. Combining sequence and southern blot analysis, the homeolog of *Exp1 *in the A-subgenome of Hai7124 was colonized to a type resembling that of the D-subgenome via nonreciprocal homoeologous exchange [23].

**Table 1:** Names and characteristics of fiber development-related genes

Gene	Accession code	Potential function
*14-3-3L*	DQ402076	14-3-3-like, may participate in the regulation of fiber elongation.
*CAP*	AB014884	adenylyl cyclase associated protein, may play a functional role during early stages of cotton fiber development.
*CEL*	AY574906	endo-1,4-beta-glucanase, necessary for plant cellulose biosynthesis.
*CelA1*	GHU58283	cellulose synthase
*CelA3*	AF150630	cellulose synthase catalytic subunit
*CIPK1*	EF363689	calcineurin B-like (CBL) protein-interacting protein kinases, was highly expressed in the elongating phase in developing fiber
*Exp1*	DQ204495	alpha-expansin 1.
*Exp*	DQ060250	Expansin, directly modify the mechanical properties of cell walls, enable turgor-driven cell extension, and likely affect length and quality of cotton fibers.
*ACT1*	AY305723	actin1, plays a major role in fiber elongation.
*BG*	DQ103699	β-1,4-glucanase, plays an important role in the loosing of the primary cell wall and in the promotion of secondary cell wall synthesis.
*ManA2*	AY187062	beta-mannosidase, glycosyl hydrolase
*Pel*	DQ073046	pectate lyase, exclusively degrade the de-esterified pectin, may play an important role in the process of normal fiber elongation in cotton.
*POD2*	AY074794	bacterial-induced peroxidase
*RacA*	DQ667981	small GTPase gene, might play an important role in the early stage of fiber development.
*RacB*	DQ315791	small GTPase gene, might play an important role in the early stage of fiber development.
*Sus1*	U73588	sucrose synthase, play an important role in the initiation and elongation of cotton fiber by inﬂuencing carbon partitioning to cellulose synthesis.
*LTP3*	AF228333	Lipid transfer protein gene, involved in cutin synthesis during the fiber primary cell wall synthesis stage

The lengths of the genomic DNA sequences isolated from the four species varied from 1020 bp (*Exp1*) to 6126 bp (*CelA3*) (Table 2). Based on the alignments between the genomic DNA and the cDNA sequences in orthologs, twelve genes (70.59%) shared the same intron/exon structures in different genomes, and variations in length were mainly caused by insertion/deletion events within introns (Additional file 2: Supplemental Figure S1A). The remaining five genes (29.41%), *CIPK1*, *CAP*, *BG*, *ManA2 *and *CelA3*, produced some structure differences caused by different exon length or numbers (Additional file 2: Supplemental Figure S1B).

**Table 2:** Structure analysis for orthologs of fiber development genes in four cotton species

Gene	Numbers of exon	Length of ORF(bp)/numbers of derived amino acids
*14-3-3L*	7	762/253
*CAP*	A_1_, Ath and Atb: 10; D_5_, Dth and Dtb: 9	A_1_, Ath and Atb: 1416/471; D_5_, Dth and Dtb: 1338/445
*CEL*	6	1860/619
*CIPK1*	1	A_1_, Ath and Atb: 1341/446; D_5_, Dth and Dtb: 1347/448
*BG*	Dth: 7; Dtb: 8; A_1_, D_5_, Ath and Atb: 9	Dth: 1050/349; Dth: 1365/454; A_1_, D_5_, Ath and Atb: 1884/627
*Exp1*	3	777/258
*Exp*	3	780/259
*POD2*	3	984/327
*RacA*	8	636/211
*RacB*	7	588/195
*Sus1*	12	2418/805
*Pel*	3	1236/411
*ManA2*	Ath: 11; Atb: 9; A_1_, D_5_, Dth and Dtb: 11	Ath: 2925/974; Atb: 1380/459; A_1_, D_5_, Dth and Dtb: 2931/976
*CelA1*	12	2925/974
*CelA3*	Ath and Atb: 8; A_1_, D_5_, Dth and Dtb: 14	Ath and Atb: 2055/684; A_1_, D_5_, Dth and Dtb: 3204/1067
*ACT1*	4	1134/377
*LTP3*	1	363/120

A_1 _= *G. herbaceum *L. var. *africanum*, D_5 _= *G. raimondii *Ulbr, Ath = A subgenome of *G. hirsutum *L. acc. TM-1, Dth = D subgenome of *G. hirsutum *L. acc. TM-1, Atb = A subgenome of *G. barbadense *L. cv. 7124, Dtb = D subgenome of *G. barbadense *L. cv. 7124.

Seventeen gene trees were constructed using the NJ method to distinguish the duplicated genes independent of evolution or local interlocus recombination after tetraploid formation. Two major clades, one including *G. herbaceum *and the A-subgenomes of TM-1 and Hai7124, the other including *G. raimondii *and the D-subgenomes of TM-1 and Hai7124, were formed for 15 genes (Additional file 3: Supplemental Figure S2A). High bootstrap values supported duplicated genes independent of evolution after tetraploid formation. Two genes were determined to have local interlocus recombination or colonization after tetraploid formation (Additional file 3: Supplemental Figure S2B). *ACT1 *from *G. raimondii *was more closely related to *ACT1s *from *G. herbaceum *and the A-subgenomes than with *ACT1s *from the D-subgenomes. This relationship suggests that *ACT1s *from the D-subgenomes evolved at an accelerated rate, relative to *ACT1s *from the A-subgenomes. The *Exp1 *sequence in Hai7124 was closer to that found in *G. raimondii*.

To locate all 17 homeolog gene pairs on our backbone genetic map [23], the subgenome-specific PCR primers (Additional file 4: Supplemental Table S2) and single-nucleotide amplified polymorphisms (SNAP) primers (Additional file 5: Supplemental Table S3) were used to detect polymorphisms between TM-1 and Hai7124. Polymorphic primer pairs were also used to survey 138 individuals of the ([(TM-1×Hai7124)×TM-1]) BC_1 _mapping population (Table 3). Eight gene pairs were located on their corresponding homeologous chromosomes, and each of six pairs were located on one of their homeologous chromosomes, while three pair of genes, *Exp*, *Exp1 *and *CelA1 *could not be mapped because no available polymorphic loci were found between TM-1 and Hai7124. A large body of data was compiled by integrating previously reported cotton fiber quality quantitative trait locus (QTL)[24-34] with the 22 identified fiber quality-related genes within 20 cM. Most genes had at least one fiber quality QTL; some had several (Table 3), indicating important fiber quality roles.

**Table 3:** Integration analysis of chromosomal locations of genes and fiber quality QTL reported in other studies

Gene	Subgenome	Chromosomal location	QTL associated with specific chromosome^a^
*14-3-3L*	At	A5	FE^h^;FL^d, i, j^;FF^d, g, i, j^;FS^d^
	Dt	D5	FF^i^;FU^i^;FL^j^;FE^g^
*CAP*	At	A13	-
	Dt	D13	FL^b, g^; FS^g^
*CEL*	At	-	-
	Dt	D6	FL^d, h, j^; FF^h, i, j, k^;FE^k^;FS^d^
*CIPK1*	At	-	-
	Dt	D7	FS^f^
*BG*	At	A13	-
	Dt	D13	-
*POD2*	At	A3	FE^b, j^; FF^b^; FS^b, h^; FL^h^
	Dt	D2	FE^j^
*RacA*	At	A12	FL^l, e, j^;FU^l^; FF^g^
	Dt	D12	FS^e^;FL^d^
*RacB*	At	A5	-
	Dt	D5	FF^i^;FU^i^;FL^j^;FE^g^
*Pel*	At	A3	FE ^b,j^; FF^b,d^; FS^b,h^;FL^h,d^; FE^j^
	Dt	-	-
*ManA2*	At	A13	-
	Dt	D13	FL^b,g,h^; FE^h^; FS^d,g^;FF^d^;FU^d^
*CelA3*	At	A8	FS^h^;FE^h^
	Dt	D8	FF^g,j^;FS^j^;FE^j^
*Sus1*	At	A5	FE^h^;FL^d,i,j^;FF^d,g,i,j^;FS^d^
	Dt	-	-
*LTP3*	At	A10	FL^b,d^; FF ^d,l^;FE^d^;FU^d^
	Dt	-	-
*ACT1*	At	-	-
	Dt	D11	FE^k^; FS^k^; FU^k^

^a ^FE Elongation; FF fineness; FL length; FS strength; FU uniformity

^b ^Frelichowski et al. (2006), ^c ^He et al. (2007), ^d^Lacape et al. (2005), ^e ^Lin et al. (2005), ^f ^Luan et al. (2009), ^g ^Qin et al. (2008), ^h ^Shen et al. (2005), ^i ^Shen et al. (2006), ^j ^Shen et al. (2007), ^k ^Wang et al. (2006), ^l ^Zhang et al. (2009) [24-34].

### Rates of sequence evolution

With only a few exceptions, purifying selection, as indicated by Ka/Ks < 1, appears to be in place for most of pairwise comparisons (Table 4). The exceptions include *G. herbaceum *(A_1 _genome, A_1_) vs *G. hirsutum *A-subgenome (Ath) of *POD2 *and D_5 _vs Dth of *RacA*, which demonstrated positive selection (Ka/Ks > 1), as well as D_5 _vs Dtb of *RacA*, A_1 _vs Atb of *14-3-3L*, A_1 _vs Ath and A_1 _vs Atb of *CAP*, which had exceptionally strong positive selection (Ka/Ks >> 1).

**Table 4:** Synonymous and nonsynonymous substitution rates in various comparisons among different cotton species

Gene	Ka/Ks/Ka:Ks ratio						
	
	A_1 _VS D_5_	A_1 _VS Ath	D_5 _VS Dth	A_1 _VS Atb	D_5 _VS Dtb	Ath VS Dth	Atb VS Dtb
*BG*	0.0081/0.0360/0.2243	9.75E-05/0.0098/0.001	0.0025/0.0362/0.0694	1.24E-05/0.0124/0.001	0.0029/0.0135/0.2127	0.0048/0.0282/0.1694	0.0076/0.0403/0.1875
*Pel*	0.0021/0.0910/0.0228	0.0024/0.0204/0.1198	0.0021/0.0041/0.5064	0.0012/0.0111/0.1106	0.0021/0.0041/0.5064	0.0022/0.0542/0.0398	0.0032/0.0912/0.0348
*ManA2*	0.0061/0.0218/0.2798	0.0041/0.0137/0.2960	0.0031/0.0085/0.3613	0.0040/0.0164/0.2419	0.0034/0.0076/0.4475	0.0049/0.0177/0.2769	0.0077/0.0183/0.4179
*POD2*	0.0119/0.0218/0.5457	0.0075/0.0069/1.0812	0.0113/0.0164/0.6852	0.0086/0.0195/0.4392	0.0131/0.0192/0.6829	0.0158/0.0313/0.5065	0.0131/0.0349/0.3753
*CIPK1*	0.0076/0.0308/0.2460	0.0026/0.0130/0.2040	0.0032/0.0134/0.2348	0.0035/0.0083/0.4179	0.0030/0.0117/0.2563	0.0077/0.0344/0.2240	0.0074/0.0247/0.3009
*RacA*	0.0117/0.0417/0.2811	0.0020/0.0061/0.3270	0.0093/0.0052/1.7812	NA/NA/NA ^a^	0.0067/0.0001/50	0.0143/0.0402/0.3554	0.0096/0.0391/0.2467
*RacB*	1.49E-05/0.0149/0.001	NA/NA/NA ^a^	NA/NA/NA ^a^	NA/NA/NA ^a^	NA/NA/NA ^a^	1.49E-05/0.0149/0.001	1.49E-05/0.0149/0.001
*EXP*	0.0018/0.0372/0.0482	0.0017/0.0335/0.0496	0.0034/0.0259/0.1305	NA/NA/NA ^a^	0.0034/0.0259/0.1305	0.0033/0.0064/0.5184	0.0016/0.0408/0.0394
*Exp1*	0.0156/0.0562/0.2784	0.0038/0.0166/0.2269	0.0032/0.0221/0.1457	0.0101/0.0650/0.1548	0.0085/0.0174/0.4899	0.0120/0.0607/0.1977	NA/NA/NA^a^
*14-3-3L*	0.0025/0.0299/0.0820	5.08E-06/0.0051/0.001	0.0039/0.0109/0.3589	0.0019/3.74E-05/50	0.0047/0.0233/0.2012	0.0024/0.0407/0.0590	0.0046/0.0036/1.2832
*CAP*	0.0144/0.0477/0.3016	0.0029/5.93E-05/48.97	0.0036/0.0063/0.5721	0.0019/3.84E-05/50	0.0059/0.0065/0.9093	0.0129/0.0437/0.2961	0.0143/0.0423/0.3369
*CEL*	0.0032/0.0270/0.1171	0.0014/0.0058/0.2392	7.80E-06/0.0078/0.001	0.0009/0.0038/0.2293	0.0007/0.0051/0.1342	0.0029/0.0400/0.0724	0.0028/0.0275/0.1030
*Sus1*	0.0045/0.0318/0.1420	0.0013/0.0054/0.2425	0.0015/0.0070/0.2072	0.0020/0.0039/0.5236	6.31E-06/0.0063/0.001	0.0053/0.0253/0.2079	0.0013/0.0054/0.2425
*CelA1*	0.0028/0.0406/0.0689	0.0005/0.0097/0.0479	0.0005/0.0027/0.1965	1.06E-05/0.0106/0.001	4.40E-06/0.0044/0.001	0.0038/0.0403/0.0939	0.0027/0.0443/0.0612
*CelA3*	0.0066/0.0406/0.1615	0.0035/0.0074/0.4772	0.0027/0.0284/0.0967	0.0043/0.0069/0.6208	0.0037/0.0219/0.1683	0.0025/0.0230/0.1067	0.0050/0.0264/0.1904
*LTP3*	0.0631/0.0752/0.8401	2.37E-05/0.0237/0.001	NA/NA/NA ^a^	1.00E-05/0.0100/0.001	NA/NA/NA ^a^	0.0610/0.0858/0.7111	0.0620/0.0667/0.9289
*ACT1*	0.0013/0.0445/0.0284	0.0012/0.0088/0.1381	4.43E-05/0.0443/0.001	0.0012/0.0088/0.1381	4.08E-05/0.0408/0.001	6.53E-05/0.0653/0.001	5.37E-05/0.0537/0.001

Letter designations are the same as in Table 2.

^a ^no synonymous and nonsynonymous site.

Because there were no nucleotide substitutions in A_1 _vs Atb of *RacA*, A_1 _vs Atb of *EXP*, D_5 _vs Dth and D_5 _vs Dtb of *LTP3*, and all *RacBs *pairs, it was not possible to compare their evolutionary rates. Although those particular "not applicable" comparisons (NA vs NA) were excluded from our analysis, the "NAs" were considered zero when they were compared with others whose Ks were not NA. In all pairwise comparisons of nucleotide diversity for each gene between subgenomes within a species [32 pairs, 16 in TM-1 and 16 in Hai7124 (*RacB *was excluded)], 62.5% (10 in TM-1 and 10 in Hai7124) had a higher evolutionary rate in the D-subgenome than in the A-subgenome. Furthermore, in the 16 gene pairs (excluding *RacB*) from the A-subgenomes of TM-1 and Hai7124, 62.5% (10 out of 16) had a higher evolutionary rate in TM-1 than in Hai7124, 31.25% (5 of 15) and were reversed and 6.25% (1 of 16, *ACT1*) showed an equivalent evolutionary rate between TM-1 and Hai7124. Similarly, in the 15 gene pairs (*RacB *and *LTP3 *were excluded) from the D-subgenomes of TM-1 and Hai7124, 60% (9 of 15) had a higher evolutionary rate in TM-1 than in Hai7124, 26.67% (4 of 15) and were reversed and 13.33% (2 of 15, *Pel *and *Exp*) showed an equivalent evolutionary rate between TM-1 and Hai7124.

Phylogenetic relationships are reflected in the nucleotide substitution results (Additional file 3: Supplemental Figure S2). Based on branch length, all of homeologs from the two tetraploid species had unequal rates of sequence evolution following allopolyploid formation. The rates at which the deviations occurred in allopolyploids are sufficient to generate branch length inequality between the A- and D-subgenomes [35].

Ka/Ks ratio comparisons showed that selection had altered the molecular evolutionary rate of some genes due to allopolyploid formation. Four genes, *Pel*, *RacA*, *Exp *and *Sus1*, in TM-1, and five genes, *Pel*, *CIPK1*, *14-3-3L*, *CAP *and *CelA3*, in Hai7124, yielded higher Ka/Ks ratios in A-At, D-Dt and At-Dt comparisons than in the A-D comparison, indicating that selection for some genes related with fiber development had acted at the tetraploid level.

### Differential expression fiber development genes

**Figure 1:**
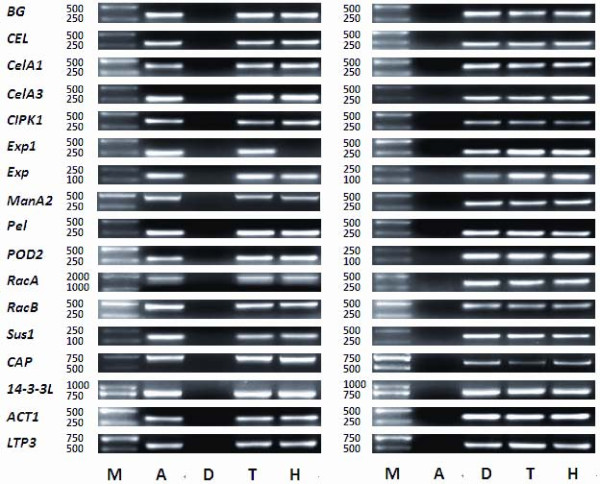
**Amplification products in four cotton species using subgenome-specific qPCR primer pairs. First line includes amplified results from A-genome specific primers; second line includes amplified results from D-genome specific primers**. "M" represents marker, "A" represents *G. herbaceum* var. *africanum*, "D" represents *G. raimondii*, "T" represents G. *hirsutum* acc. TM-1, "H" represents G. *barbadense* cv. Hai7124. Numbers represent the sizes of the makers (bp).

After the specificity of homeolog-specific primer pairs were confirmed by PCR amplification of genomic DNA from *G. herbaceum *(A-genome), *G. raimondii *(D-genome), TM-1 and Hai7124 (Figure 1), their homeolog transcripts in young tetraploid cotton fiber were further detected by qPCR analysis. The relative expression values at 10 different fiber development stages were obtained by combining the homeolog transcripts of each gene at the same stage. Expression for the 17 genes could be broken down into five categories (Additional file 6: Supplemental Figure S3): 1) fiber initiation and early elongation (0-8 DPA), such as *Exp*, *POD2 *and *ManA2 *(Additional file 6: Supplemental Figure S3A); 2) fiber elongation (3-17 DPA), such as *Exp1*, *Pel*, and *LTP3 *(Additional file 6: Supplemental Figure S3B); 3) primary-secondary transition period (17-23 DPA), such as *BG*, *CEL *and *CelA1 *(Additional file 6: Supplemental Figure S3C); 4) both at fiber initiation and early elongation period (0-8DPA) and secondary cell wall thickening period (20-23DPA), such as *Sus1*, *14-3-3L *and *RacB *(Additional file 6: Supplemental Figure S3D); 5) the whole fiber developmental period, such as *CelA3*, *CAP*, *ACT1*, *RacA *and *CIPK1 *(Additional file 6: Supplemental Figure S3E). In the last category, however, transcript preference was shown at some stages. For example, *CelA3 *and *CAP *were expressed preferentially at the fiber elongation and secondary cell wall thickening stages (8-23 DPA), but had moderate expression at 0-5 DPA.

Gene expression differences in TM-1 and Hai7124 were further clarified by statistical analysis of least signification difference (LSD). Greater expression in Hai7124 than in TM-1 was observed for *14-3-3L *except at 20 DPA, and for *CelA3 *except at 5, 17 and 23 DPA. Other gene transcripts showed different expression advantages in the two cotton species at various fiber developmental stages.

At fiber initiation and early elongation (0-8 DPA), most genes, including *Exp, ManA2, Sus1, RacB, CelA3, CAP *and *RacA*, had significantly higher expression levels in Hai7124 than in TM-1. During fiber elongation (5-14 DPA), the expression profiles of genes preferentially expressed during that period were either biased to TM-1 or Hai7124 or were equally expressed between the two. Five genes, *Exp1*, *Pel*, *CAP*, *CIPK1 *and *RacA*, were expressed preferentially in TM-1 or equally between TM-1 and Hai7124, except at 8 DPA, that these same genes showed significantly greater expression levels in Hai7124; *LTP3 *and *ACT1 *showed significantly higher expression levels in TM-1 than in Hai7124; expression of *CelA3 *was higher in Hai7124.

During primary-secondary cell wall transition (17-23 DPA), peak expression occurred earlier in TM-1 than in Hai7124 for most genes (*CAP*, *CelA3*, *CIPK1*, *CEL, BG, RacB*, *Sus1 *and *14-3-3L*). *ACT1 *and *RacA *expressed equally in TM-1 and Hai7124 at 17 and 20 DPA, but significantly greater in Hai7124 at 23 DPA. The extended fiber development period, as indicated by higher expression at a later DPA, may help explain why *G. barbadense *has an extra long staple cotton. One gene, *CelA1*, showed no significant expression difference between TM-1 and Hai7124.

### Genome-specific expression of the homeologs

Based on the homeolog expression profile, 17 diagnostic genes in TM-1 and 17 in Hai7124 were further evaluated. Of the 34 genes, 32.35% (11) were equally expressed between the A- and D-subgenomes, 41.18% (14) were A-subgenome biased, 20.59% (7) were D-subgenome biased and 5.88% (2) were A- or D-biased at different stages.

**Figure 2:**
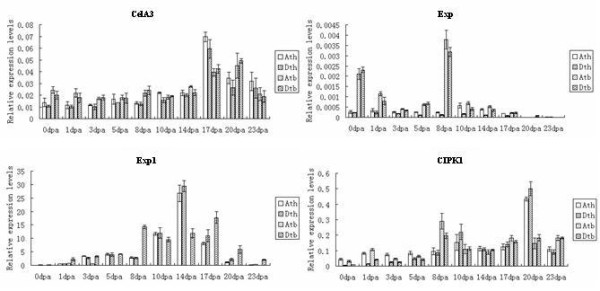
**Q-PCR analysis for homeologous expression of genes expressed equally between A- and D-subgenomes**. Significant values were obtained by comparison between the two subgenomes. * P < 0.05, ** P < 0.01. See Table 2 for abbreviation designations. Vertical bars represented standard deviation (STD).

The 17 fiber development genes were clustered into three comparison patterns between TM-1 and Hai7124. First, homeologs for *CelA3*, *Exp*, *Exp1 *and *CIPK1 *in both TM-1 and Hai7124 were equally expressed between the A- and D-subgenomes in the preferentially-expressed stages (Figure 2). Of these, *Exp1 *had equal transcript levels from the two homeologs in TM-1 and Hai7124, with two distinguishable copies in TM-1 and two undistinguishable copies in Hai7124. These data were consistent with the fact that the duplicated loci for *Exp1 *in Hai7124 had the same sequence as the D-subgenome (Figure 2).

**Figure 3:**
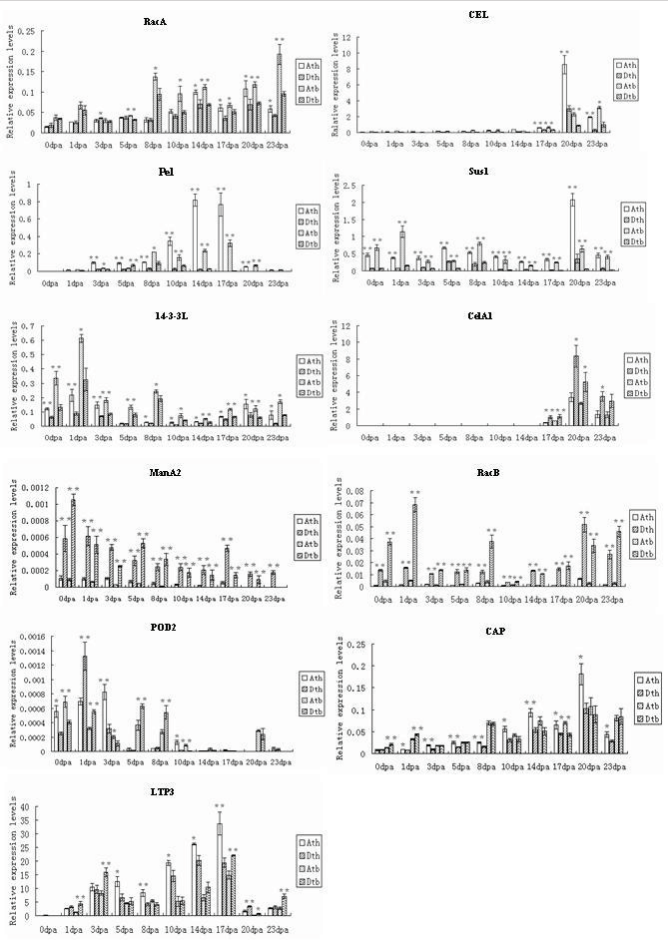
**Q-PCR analysis for homeologous expression of genes with A or D-subgenome biased expression**. Significant values and vertical bars were same with Figure 2.

Second, the transcripts of 11 genes, *CEL*, *Pel*, *Sus1*, *14-3-3L*, *RacA*, *CelA1*, *ManA2*, *RacB*, *CAP*, *LTP3 *and *POD2*, were A- or D-subgenome biased (Figure 3). Among these, *CEL*, *Pel*, *Sus1*, *14-3-3L *and *RacA *were A-subgenome biased and *CelA1*, *ManA2 *and *RacB *were D-subgenome biased in both TM-1 and Hai7124 at all stages. The transcripts of the homeologs of *CAP*, *LTP3 *and *POD2 *were significantly altered in the preferentially expressed stages in TM-1 and Hai7124. In TM-1, the transcripts of *CAP *and *LTP3 *were significantly A-subgenome biased. However, the transcripts of the two genes in Hai7124 were equivalently expressed at most stages, only D-subgenome bias in *LTP3 *in the primary-secondary cell wall transition period detected. Expression of *POD2 *was A-subgenome biased at 0, 3 and 10 DPA and D-subgenome biased at 1 DPA in TM-1. In Hai7124, *POD2 *expression was A-subgenome biased at 0, 3 and 10 DPA and D-subgenome biased at 1, 5 and 8 DPA.

**Figure 4:**
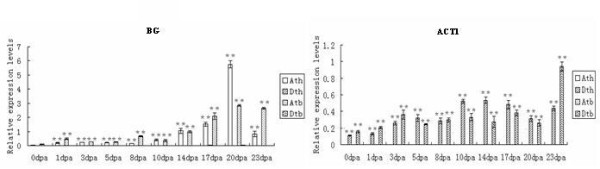
**Q-PCR analysis for homeologous expression of genes with subgenome-specific expression**. Significant values and vertical bars were same with Figure 2.

Third, *BG *was significantly (P < 0.001) affected only from the A-subgenome, and *ACT1 *was significantly (P < 0.001) affected from the D-subgenome at all stages in both TM-1 and Hai7124 (Figure 4). Based on the comparison patterns and the structural analysis of the two genes, we proposed that the homeolog of *BG *from the D-subgenome might be silenced and that of *ACT1 *from the A-subgenome may have novel roles in other species (neofunctionalization).

Differences between TM-1 and Hai7124 in transcriptome contributions of the subgenome at key fiber developmental stages were detected. During initiation and early elongation of the fiber, 10 gene transcriptions showed greater expression levels in Hai7124 than in TM-1. Of those, the D-subgenome contributed higher amounts of *ACT1*, *RacB *and *Man2*, while the A-subgenome contributed higher amounts of *Sus1*, *CIPK1 *and *14-3-3L. CelA3*, *Exp*, *CAP *and *RacA *were equally supplied by both subgenomes.

At 8 DPA, corresponding to the close of fiber plasmodesmate [36], the transcriptions of 12 genes (*Exp1*, *Pel*, *POD2*, *CelA3*, *BG*, *Sus1*, *CAP*, *Exp*, *RacA*, *RacB*, *14-3-3L *and *CIPK1*) were sharply accumulated in Hai7124. Of those, the transcripts of *RacB *and *POD2 *were contributed mainly from D-subgenome, that of *BG*, *Pel*, *Sus1 *and *RacA *from A-subgenome and others by both A- and D-subgenomes.

At the primary-secondary transition period, the expression of 10 genes, *CelA3*, *CAP*, *ACT1*, *RacA*, *CIPK1*, 14-3-3L, *Sus1*, *RacB*, *CEL *and *BG*, occurred earlier in TM-1 than in Hai7124. The transcripts of *ACT1 *and *RacB *were mainly from D-subgenomes; those of *BG*, *CEL *and *Sus1 *were mainly from A-subgenomes, and other genes were from both A- and D-subgenomes. Based on these data we inferred that the expression accumulation of the A-subgenome, or the combination of A- and D-subgenomes, played critical roles in fiber quality divergence of *G. hirsutum *and *G. barbadense*. However, the expression of D-subgenome alone also played an important role.

## Discussion

### Evolutionary fate of duplicated genes

For each gene that was studied, allopolyploid species should have two homelogs, representing descendants from the A-genome and D-genome donors at the time of polyploidy formation. Cronn et al. [35] indicated that most duplicated genes in allopolyploid cotton evolved independently of each other. Our phylogenetic analyses support this hypothesis, and the independent evolution of several genes was distinctively evident in their structure, in our study. For example, *CAPs *and *RacBs *had the same structure between each diploid and its counterpart in allopolyploid cotton (A- and At-subgenome, D- and Dt-subgenome), but the different structures were apparent in the A-D comparison (Additional file 2: Supplemental Figure S1B). Though expression of *ManA2 *from the At-subgenome of Hai7124 ceased rather early in the growth process, the structure difference between the A-, At-subgenomes and D-, Dt-subgenomes was also distinct. The fact that the structure of the At- and Dt-subgenomes mirrored their putative ancestral diploid species suggested the difference may have occurred before allopolyploid formation and evolved independently in allopolyploid cotton. *CelA3s *from At-subgenome of TM-1 and Hai7124 displayed the same mutation, which altered their coding regions, indicating not only independent evolution, but also parallel evolution between TM-1 and Hai7124. This change, however, was not detected in their putative ancestral diploid species, suggesting accelerated evolution of *CelA3 *in the At-subgenome after allopolyploid formation. Though most genes independently evolved in allopolyploid cotton, there were some exceptions. For example, *Exp1 *from At-subgenome were colonized in Hai7124 by Dt-subgenomes.

Relative to expression, duplicate genes can follow one of three evolutionary paths. First, one copy may evolve into a nonfunctional pseudogene [37-41]. Second, the multiple copies can contribute to an increase in the gene expression level [42,43] or both copies can suffer mutations but the combined action of both gene copies is necessary to maintain original function and expression levels (subfunctionalization) [40,44,45]. Third, one copy may gain a novel beneficial function (neofunctionalization) that is selectively maintained within the genome [40,46-48]. We measured homeolog-specific contributions to the transcriptome in allopolyploid cotton fiber by Q-PCR analysis. Because the majority (64.70%) of diagnostic genes exhibited subgenome-specific bias to the A or D-subgenome, subgenome-biased expression in cotton fiber developmental stages was considered commonplace. This result was consistent with previous studies [49-55]. Most of genes in our study exhibited the same expression bias in the two cultivated cotton species, TM-1 and Hai7124. However, some inconsistencies were detected in three genes (*CAP, LTP3 *and *POD2*), suggesting that these genes may have had different roles in the interspecific divergence between *G. hirsutum *and *G. barbadense*. Artificial selection by humans of certain desirable fiber traits may have also influenced *G. hirsutum *and *G. barbadense *genetic structure [55].

Synthesizing structures and expression profiles of the duplicates, their possible fates are inferred. *BG *accumulated solely in A-subgenome transcripts (D-subgenome silenced), in both TM-1 and Hai7124 (Figure 4). *BG*s obtained from the D-subgenomes of TM-1 and Hai7124 had a nucleotide deletion and a nonsense mutation, respectively, which altered the ORFs (Table 2). The structure difference suggests that *BG *in the D-subgenomes of TM-1 and Hai7124 may be pseudogenes. On the other hand, while *CAP *and *CelA3 *had different A- and D-subgenome structures in both TM-1 and Hai7124 (Additional file 2: Supplemental Figure S1B), their A- and D-subgenome expression profiles were active (Figure 2, 3). Therefore, the duplicated genes of *CAP *and *CelA3 *may be subfunctionalized. Similarly, the functions of duplicated genes from *CEL*, *Sus1*, *14-3-3L*, *RacA*, *RacB, Exp*, *Exp1*, *CIPK1*, *CelA1*, *Pel*, *ManA2*, *LTP3 *and *POD2 *were also subfunctionalized (Figure 3). Because *ACT1 *transcripts of A subgenomes could not be detected at all stages of fiber development (Figure 4), they may have evolved new functions.

### Domestication of allopolyploid cotton

Numerous plant species have been selectively bred over the course of human social evolution [56]. Allopolyploid cotton species are believed to have formed about 1-2 million years ago, by hybridization between a maternal Old World diploid A-genome *G. herbaceum *[57] and paternal New World diploid D-genome *G. raimondii *[57-59]. The allotetraploid lineage gave rise to five extant tetraploid species, including *G. barbadense *and *G. hirsutum*, known for their superior fiber quality and high yield, respectively. In the present study, the Ka/Ks ratios among four cotton species indicated that selection of fiber development genes occurred at the tetraploid level. By comparing the nucleotide diversity between TM-1 and Hai7124 within the same subgenome, most genes (62.5% in A-subgenome and 60% in D-subgenome) had a higher evolutionary rate in TM-1 than in Hai7124, which may be associated with longer and more frequent cultivation of TM-1. Given these data, we propose that diversity evolution between A- and D-subgenomes within a species or between TM-1 and Hai7124 within the same subgenome was due to both natural and artificial selection pressure [55].

### Gene expression differences between TM-1 and Hai7124

*G. hirsutum *and *G. barbadense *are two domesticated cotton species possessing very different agronomic and fiber quality characteristics with *G. barbadense *having superior fiber quality. Rapp et al. (2010) studied the transcriptomes of cotton fibers from wild and domesticated accessions (*G. hirsutum*) and found that human selection during the initial domestication and subsequent crop improvement had resulted in a biased upregulation of components of the transcriptional network during fiber development [60]. In this study, of the 17 fiber development-related genes, 14 had the similar expression pattern and three that did not, in TM-1 and Hai7124 (Figure 2, 3, 4). Of three genes, the transcripts of homeologs were significantly A- or D-subgenome biased in TM-1. However, in Hai7124, homeolog transcripts were equally expressed between the two subgenomes or D-subgenome biased. Though 14 genes had the same expression patterns between TM-1 and Hai7124, the relative expression levels were different at most stages. While the same A- or D-biased or equal expression profile in the two cultivated cotton species might be related to functional partitioning of genomic contributions during cellular development after allopolyploid formation, significant alternation of homoelog A/D ratio and expression difference at the same fiber developmental time points between *G. hirsutum *and *G. barbadense *indicated that domestication for different fiber qualities may play an important role in fiber quality divergence of *G. hirsutum *and *G. barbadense*.

In previous study, fiber growth curves have shown longer fiber elongation phases in domesticated *G. hirsutum *than that in wild *G. hirsutum*, and further comparative gene expression profiling of isolated cotton fibers over a developmental time course of fiber differentiation indicated that domesticated TM-1 displayed a much higher level of transcriptional variation between the sampled time points than the wild accession did [60]. In the study, the expression peak of transcripts in most genes was earlier in TM-1 than in Hai7124, especially at the primary-secondary transition period, which indicated that most genes related to fiber development expressed longer and more intensely in Hai7124. Resulting differences in mRNA levels may lead to changes in enzyme activity, further contributing to phenotypic differences between the two cotton species. Several genes that are differentially expressed in TM-1 and Hai7124 should be further mined.

The 14-3-3 protein is an important regulatory protein. Shi et al. [6] proposed that the *Gh14-3-3L *transcripts are highly accumulated during early cotton fiber development, suggesting that *Gh14-3-3L *may be involved in regulating fiber elongation. Our data showed that, although *14-3-3L *is expressed preferentially in the early development stages of cotton fibers in both TM-1 and Hai7124, the relative expression values were significantly different. The expression of *14-3-3L *was significantly higher in most stages in Hai7124 (Additional file 6: Supplemental Figure S3E), than in TM-1. Furthermore in the primary-secondary transition period of fiber development, a secondary expression peak of *14-3-3L *was also detected, indicating *14-3-3L *may also be involved in thickening of the secondary cell wall, a function that has been reported in other species [61]. At 23 DPA, the expression of *14-3-3L *decreased in TM-1, but was still increasing in Hai7124, suggesting a longer transcriptional period.

*CelA1 *was solely expressed during secondary wall cellulose synthesis, with no significant expression difference between TM-1 and Hai7124 (Additional file 6: Supplemental Figure S3C). The importance of *CelA *genes in secondary wall cellulose synthesis in developing cotton fibers has been reported by other researchers [16]. Expression of *CelA3 *at 17 DPA was significantly higher in TM-1 than in Hai7124. However, strength dropped much faster in TM-1 (e.g., at 20 DPA, Additional file 6: Supplemental Figure S3E) than in Hai7124 after that, which more stable expression was detected over a longer period of time in Hai7124.

*Rac *genes (coding for GTP-binding Rac proteins) are involved in various physiological processes, including cell polar growth [62-65], synthesis of the secondary wall [66-68], resistance response [69,70] and signal transduction [71-73]. In the present study, *RacA*s were predominantly expressed at the fiber elongation and secondary wall synthesis stages (Additional file 6: Supplemental Figure S3E), indicating that the role of *RacA *may be similar to that of *Rac13*, which is involved in the signal transduction pathway for cytoskeleton organization [66]. Similarly, *RacB *was predominantly expressed at the secondary wall synthesis stage in TM-1, and preferentially expressed at the initiation of fiber development and secondary wall synthesis in Hai7124 (Additional file 6: Supplemental Figure S3D), suggesting multiple roles for *RacB*. Because *RacA *and *RacB *expressions were consistently higher at most stages in Hai7124 relative to TM-1, the two genes may be contributing to fiber quality diversity.

Sucrose synthase (Sus) affects initiation and elongation of the single-celled fibers, and also has a major role in cell wall cellulose synthesis [9,74-77]. Cotton fiber cell initiation and elongation are very sensitive to changes in *Sus *activity [9]. The expression peak of cotton sucrose synthase genes transcripts was earlier in wild cotton than in TM-1[60]. Our study showed that greater *Sus1 *expression in Hai7124 than in TM-1 at initiation (0 and 1 DPA) and 8 DPA and at 3 and 5 DPA was reversed. At 20 DPA, *Sus1 *expression was significantly higher in TM-1, possibly as a result of earlier termination of fiber elongation and earlier initiation of cell wall synthesis in TM-1.

Recent studies of comparative gene expression profiling of isolated cotton fibers [60,78] have identified genes controlling differences in fiber growth between wild and domesticated cotton [e.g., genes encoding tubulin isoforms, endotransglycosylase/hydrolases, cytochrome P(450) monooxygenase, and antioxidant enzymes]. These studies will provide new clues in the future studies to better understand the interspecific divergence of fiber development in the two cultivated tetraploid cotton species, *G. barbadense *and *G. hirsutum*.

### Putative role of D-subgenome in interspecific divergence

Most genes (62.5% in TM-1 and 62.5% in Hai7124) exhibited a higher evolutionary rate in the D-subgenome than in the A-subgenome, indicating that the D-subgenome of the allotetraploid evolved faster than the A-subgenome [59,79-82]. Considering the weight of evidence provided by genome-specific expression of homeologs, chromosome location of genes and QTL distribution for fiber qualities (Table 3), we also determined the subgenome transcriptome contribution in interspecific divergence between TM-1 and Hai7124. In the integration interval of genes and QTLs, the gene transcripts were significantly A-subgenome biased, and the QTLs associated with fiber qualities were detected in corresponding A-subgenome chromosome bins. Similarly, the gene transcripts were significantly D-subgenome biased and relative fiber QTLs were detected in corresponding D-subgenome chromosome bins. While the transcripts of *CAP *was significantly A-subgenome biased in TM-1, but equally expressed in Hai7124 in the fiber developmental period, implying an important D-subgenome contribution for elite fiber quality traits in Hai7124. Integration of *CAP *duplicated loci with fiber length and strength QTLs in D13, indicates domestication and artificial selection of tetraploid plants for superior fiber quality resulted in more significant evolutionary dynamics in the D-subgenome than in the A-subgenome.

## Conclusion

The study provided us the systematic report on the interspecific divergence for fiber development between *Gossypium barbadense *and *G. hirsutum *by analyzing structures, molecular evolution and transcripts levels of fiber development-related genes. The results indicated that selective breeding for certain desirable fiber qualities may have played an important role in divergence of the two cultivated tetraploid cotton species.

## Methods

### Plant materials

Of the 17 genes selected for this study (Table 1) were described in previous reports as being expressed in developing cotton fibers; their sequence information was deposited in GenBank. The orthologous loci of each of the 17 genes were isolated from two allotetraploid cottons, *G. hirsutum *acc. TM-1, *G. barbadense *cv. Hai7124, and living models of their two ancestral genome donors, *G. herbaceum *var. *africanum *(A-genome) and *G. raimondii *Ulbrich (D-genome). For expression analysis, developing cotton ovules and attached fibers were harvested from three replicates of TM-1 and Hai7124 on 0, 1, 3, 5, 8, 10, 14, 17, 20 and 23 days post-anthesis (DPA). The mapping population was comprised of 138 BC_1 _individuals generated from the cross [(TM-1×Hai7124) ×TM-1] [19].

### PCR amplification, cloning and sequencing

Based on GenBank deposited sequences, gene-specific PCR primer pairs were individually designed for PCR-amplification of full-length sequences of the 17 genes. For the smaller genes, we designed one primer pair; at least two nested primer pairs were designed for the larger genes (Additional file 1: Supplemental Table S1).

Standard polymerase chain reaction (PCR) was completed using High-fidelity ExTaq DNA polymerase (TaKaRa Biotechnology (Dalian) Co., Ltd., China). The PCR products were cloned into pMD18-T Vector (TaKaRa) according to the manufacturer's instructions, and sequenced from plasmid DNA templates. In order to obtain the sequence from both the A-subgenome and D-subgenome, at least 10 clones for each gene from each of the tetraploid species, TM-1 and Hai7124, were picked randomly and sequenced. Homeologs were identified by comparison to sequences from their diploid progenitors. To avoid possible complications originating from PCR recombination in allopolyploid cotton [83], a minimum of three clones was used to determine the gene sequence in each duplicated copy. In cases where PCR recombination occurred, 10 additional clones were sequenced to verify the corresponding correct sequence [83]. When the copy number of genes was difficult to identify by sequencing, it was confirmed by southern blot analysis.

Lengths were determined using alignment analysis, which was applied to each relevant sequence of applicable gene. For genes with one primer pair, the A- and D-subgenome sequences were clustered using the Neighbor-Joining method in MEGA3.1 http://www.megasoftware.net/. For those genes having more than one primer pair, sequence segments from the same genomes and subgenomes were contiged by CAP3 software.

### Gene structure and phylogenetic analyses

Six DNA sequences for each gene, including homeologs and orthologs, isolated from the four species (A-, D-genome and the A and D subgenome from the allotetraploid), were aligned by ClustalX http://www.ebi.ac.uk/Tools/msa/clustalw2/. Based on the sequences of genomic DNA and the cDNA, the structure of each gene was illustrated by the Gene Structure Display Server (GSDS, http://gsds.cbi.pku.edu.cn/chinese.php). Phylogenetic analyses of the orthologs for each gene were performed using the Neighbor-Joining method in MEGA3.1 http://www.megasoftware.net/.

### Chromosomal location

In order to locate all 17 pairs of homeologs on our genetic map, PCR primers were designed according to the sequence size difference between the two allotetraploid cottons (Additional file 4: Supplemental Table S2). For homeologs with no size polymorphism, single-nucleotide amplified polymorphism (SNAP) primers were designed based on a SNP between the TM-1 and Hai7124 sequences at a putative locus (Additional file 5: Supplemental Table S3). SNAP is a modified allele-specific amplified method, it introduce an additional base pair change in the primer to increase the specificity of the primer [84]. All SNAP primers was designed following Drenkard et al. [84] and using SNAPER http://ausubellab.mgh.harvard.edu.

All the primer pairs exhibiting polymorphisms between TM-1 and Hai7124 were used to survey 138 individuals of the BC_1 _mapping population. The polymorphic loci were integrated in our backbone map [22] using Joinmap 3.0 software [85].

### Evolution and ratios of sequence forms

For each gene, synonymous substitution rates (Ks), nonsynonymous substitution rates (Ka) and their ratios (Ka/Ks) in various comparisons among the four species (A_1 _VS D_5_, A_1 _VS At, D_5 _VS Dt, At VS Dt) were calculated for the coding region using KaKs_Calculator http://sourceforge.net/projects/kakscalculator2/. Ks is the rate of evolution and the Ka/Ks ratio indicates the selective force acting on the protein [86]. A Ka/Ks ratio of 1 represents neutral evolution, that is, the number of nonsynonymous changes at each possible nonsynonymous site is the same as the number of synonymous changes per synonymous site. Ka/Ks < 1 indicates a purifying selection (selection generally eliminates deleterious mutations and maintains protein status quo); Ka/Ks > 1 indicates positive selection, where selection may be modifying the protein. The higher the ratio is, the stronger the evidence that selection is occurring.

### Development of homeolog-specific PCR primer pairs

Homeolog-specific primers were designed and applied based on sequence differences between duplicated loci (Additional file 7: Supplemental Table S4). Specificity of those primers was detected by PCR amplification of genomic DNA from *G. herbaceum *(A-genome), *G. raimondii *(D-genome), TM-1 and Hai7124. Primers specific for the A-genome resulted in amplifications from *G. herbaceum*, TM-1 and Hai7124, but not from *G. raimondii*, which indicated that the amplification products of TM-1 and Hai7124 were from their A-subgenomes. Conversely, the D-genome specific primers only amplified from *G. raimondii *and D-subgenome of the allopolyploid.

### RNA isolation and Q-PCR analysis

Total RNA was extracted from young fibers of *G. hirsutum *acc. TM-1 and *G. barbadense *cv. Hai7124 at 0, 1, 3, 5, 8, 10, 14, 17, 20 and 23 DPA using the CTAB-acid phenol extraction method [87]. RNA samples were treated with DNase I (Ambion, Austin, TX, USA) according to manufacturer's instructions to remove trace contaminants of genomic DNA. Total RNA samples (1 μg per reaction) were reversely transcribed into cDNAs by avian myeloblastosis virus (AMV) reverse transcriptase, and the cDNAs were used as templates in Q-PCR reactions.

For each gene, the homeolog-specific primers were designed based on the SNP between A- and D-subgenome homeologs (Additional file 7: Supplemental Table S4). A cotton elongation factor (*EF1a*) gene was used as a standard control in the Q-PCR reactions (Additional file 7: Supplemental Table S4). The Q-PCR reaction and calculated relative value for expression level of each homeolog were determined as described by Wang et al. [8]. The information of the Q-PCR analysis based on the MIQE checklist was submitted in Additional file 8.

## Abbreviations

SNP: Single Nucleotide Polymorphism; SNAP: Single-Nucleotide Amplified Polymorphisms; QTL: Quantitative Trait Loci.

## Authors' contributions

Experiments were designed by WZG with suggestions from TZZ. WZG and HYZ conceived the experiments and analyzed the results. HYZ carried out most of the experiments and all computational analyses. Q-PCR Analysis was performed by XYH. JHL, LZ, and XYX participated in part of experiments. HYZ and WZG drafted the manuscript and TZZ revised the manuscript. All authors read and approved the final manuscript.

## Supplementary Material

Additional file 1 **Table S1**. Primer pairs used for amplifying the full-length genomic sequences of each gene. Click here for file

Additional file 2 **Figure S1**. Structure analysis of fiber development-related genes in four cotton species. A: *G. herbaceum *L. var. *africanum*; D: *G. raimondii *Ulbr; TM-1-At: A subgenome of *G. hirsutum *L. acc. TM-1; TM-1-Dt: D subgenome of *G. hirsutum *L. acc. TM-1; 7124-At: A subgenome of *G. barbadense *L. cv. 7124; 7124-Dt: D subgenome of *G. barbadense *L. cv. 7124. A. Orthologs having the same structures among four different cotton species. B. Orthologs having different structures among four different cotton species. Click here for file

Additional file 3 **Figure S2**. The phylogenetic trees of 17 genes in four cotton species. Bootstrap values (%) based on 1000 replicates are indicated beside the nodes. A. Genes that evolved independently between the A- and D-subgenomes. B. Genes that evolved through different degrees of colonization between the A- and D-subgenome. Click here for file

Additional file 4 **Table S2**. PCR primer pairs used for gene location. Click here for file

Additional file 5 **Table S3**. SNP primer pairs used for gene location. Click here for file

Additional file 6 **Figure S3**. Q-PCR analysis for total expression of genes. Significant differences between TM-1 and Hai7124 at the same stages indicated by * P > 0.05, ** P > 0.01. Vertical bars represented standard deviation (STD). A. Genes expressed preferentially at fiber initiation and early elongation (0-8 DPA). B. Genes expressed preferentially at fiber elongation (3-17 DPA). C. Genes expressed preferentially at primary-secondary transition (17-23 DPA). D. Genes expressed both at fiber initiation and early elongation period (0-8DPA) and secondary cell wall thickening period (20-23DPA). E. Genes expressed coving the whole fiber developmental period. Click here for file

Additional file 7 **Table S4**. Subgenomic-specific PCR primer pairs used for Q-PCR analysis. Click here for file

Additional file 8 **The information of the Q-PCR analysis based on the MIQE checklist**. Click here for file
