# Supplementary material for: Structure, expression differentiation and evolution of duplicated fiber developmental genes in Gossypium barbadense and G. hirsutum
Source: BMC Plant Biol. 2011 Feb 25;11:40. doi: 10.1186/1471-2229-11-40 (PMC3050799; doi:10.1186/1471-2229-11-40)
Supplement: Additional file 1 — Table S1. Primer pairs used for amplifying the full-length genomic sequences of each gene. [file 1471-2229-11-40-S1.DOC]

**Table S1. Primer pairs used for amplifying the full-length genomic sequences** of each gene.

| Gene | Number of primer pairs | Primer pairs(5’-3’) | |
| --- | --- | --- | --- |
|  |  | F | R |
| *14-3-3L* | 1 | AGCGGCCATGGAGAAGGAAAGAG | GAAAGCAGGTCACTCTAGCAG |
| *CAP* | 2/3^a^ | GCGATTAGTTTGAGAATGGAG^b^ | TTGTCCACTGAAATTGTTGG^b^ |
|  |  | GTTGTTAGTGCCAGTGGAAAGG^c^ | CCGTACGGAAGAAGTAAAATTCACCC^c^ |
|  |  | AAGGAAACACATTCAAGCTC ^d^ | CCAACAATTTCAGTGGACAA^d^ |
|  |  | CCAACAATTTCAGTGGACAA^e^ | GGTTTCATCATCCCAAAATC^e^ |
| *CEL* | 2 | AGATCTGTCGGTGCTCCTGCAT | ACGCAGGACATCAGTTGAATAG |
|  |  | GTGATTATCTTGATGCTGCTGA | CGTTTTCATGGTTTCCAAGGTG |
| *CelA1* | 3 | TTGAGAATGATGGAATCTGGGGTT | CCTTCGATGTCACGAGCACCAC |
|  |  | AACTTCTTGGCCAGGAAATAAC | GCTTCGTACCCTTTGTTGAG |
|  |  | ATGGTATGGCTTTGGAGGTG | ATCAACAATCAATGGAAATGCAGC |
| *CelA3* | 4 | ATTGATATTTGGACGACCTGTT | GGCTACATTGCCTAGTCCTGAT |
|  |  | CCATATTCCTCTGCTCACCAG | ATGCCATCCAGACCCCTCAAG |
|  |  | CAGTAGAATTCCTTTTGATGC | CTTGAGCCTACCACTATAACC |
|  |  | CCCAGCCTTTAAAGGTTCTGC | CACCAAAACAAATCTTCAGTC |
| *CIPK1* | 1 | AATGGAGTCCTTCCAAATCACC | TGTGTACGCAATATCCAAACCC |
| *Exp1* | 2^f^ | CTCAAATGGCAACCAAAACGAT | CTACCTCGGCATAAAACGCTCA |
|  |  | GTCAGCCAATTGTTTGAGCTAGCTAG | TTCAGACATTATGGATATATCCTCTA |
| *Exp* | 1 | ATGGCAATGGTGAATGTAATCAGC | GAGGGGTTGCAAAATCTATCAAAC |
| *ACT1* | 1 | ATTGTAAAAGATGGCCGACGGT | CTTCCCGCACATAAACCAGACT |
| *BG* | 2 | TTGGGATTGTAAGTGAGCATC | GGTTGTTCCACCATCTCCTAC |
|  |  | TGTTGACGCAATGACAGAGAT | GGATCCCCAACATGACATAGC |
| *ManA2* | 4 | CTTCTCACTTCGCTCCCTAC | CAGCAAGTTTGAACCATCAG |
|  |  | CGAAGACATTCCCTTGAAGT | CTGTCCATTCACCTTGAACA |
|  |  | ATGGAATGGGAAAGCAGTCTC | TCCACATACGGGATGTCCAAC |
|  |  | AGCTTTATTGGAGGGTTGGAC | CCGAGTAACCAGAATCATTCA |
| *Pel* | 1 | TTGCCTCACAAAACAAGCAAAC | GCCATCATTATCATCAACAACG |
| *POD2* | 2 | TCCCACAAACTCACAATCAA | CAAGCTGAAGTCCCTATGGT |
|  |  | TGCCTGGATTTAGAGAGTCC | ATCACCCCCACGATTAACT |
| *RacA* | 2 | AATGGCTTCAAGCGCTTCAAG | GGTGAAAACAATGGAGAAAAG |
|  |  | AATTGCTTCTGCACAAGTCCT | CGACTTGATCCTGATTGTCTA |
| *RacB* | 3 | AGAATATGCCGCTCTTGTGAGAT | TCAGGGTGGATGTGTTGATGG |
|  |  | CCAAAGGTTCTTGATCTAGCTT | CAGCAGTGTCCCATAAACCT |
|  |  | GTGCAAATGTCGTCGTCGATG | ATGGAACCAACTGCCAGCTTCTC |
| *Sus1* | 3 | GTTAAGTTCAAAGAAATGGCT | TCCCTTTTCTGTTCTGAAGG |
|  |  | AATGTTTTGGGGTATCCCGAC | CATCTGAATTGGCCGTTCAAG |
|  |  | TGTAGGTGGTGATAGGCGAAAG | GGAATTGACGGGCGCACAAC |
| *LTP3* | 1 | CACCCGCTACTACTTTTGGCT | TGGATTTATCGTGCGAAGACT |

^a^ *CAPs* from A-genome and A-subgenome amplified using two primer pairs, while from D-gnome and D-subgenome amplified using three primer pairs.

^b、c^ Primer pairs used to get *CAPs* in A-genome and A-subgenome.

^b、d、e^ Primer pairs used to get *CAPs* in D-genome and D-subgenome.

^f^ Two primer pairs both for the full-length of *Exp1*.
