# Supplementary material for: Structure, expression differentiation and evolution of duplicated fiber developmental genes in Gossypium barbadense and G. hirsutum
Source: BMC Plant Biol. 2011 Feb 25;11:40. doi: 10.1186/1471-2229-11-40 (PMC3050799; doi:10.1186/1471-2229-11-40)
Supplement: Additional file 2 — Figure S1. Structure analysis of fiber development-related genes in four cotton species. A: G. herbaceum L. var. africanum; D: G. raimondii Ulbr; TM-1-At: A subgenome of G. hirsutum L. acc. TM-1; TM-1-Dt: D subgenome of G. hirsutum L. acc. TM-1; 7124-At: A subgenome of G. barbadense L. cv. 7124; 7124-Dt: D subgenome of G. barbadense L. cv. 7124. A. Orthologs having the same structures among four different cotton species. B. Orthologs having different structures among four different cotton species. [file 1471-2229-11-40-S2.DOC]

**A**


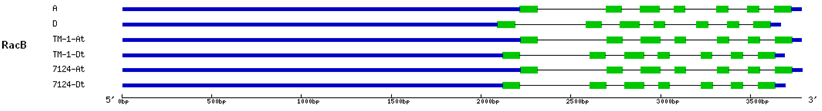

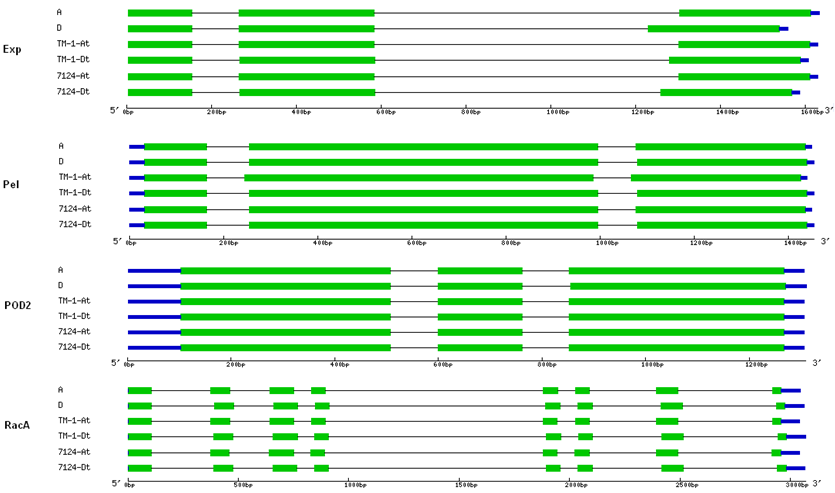

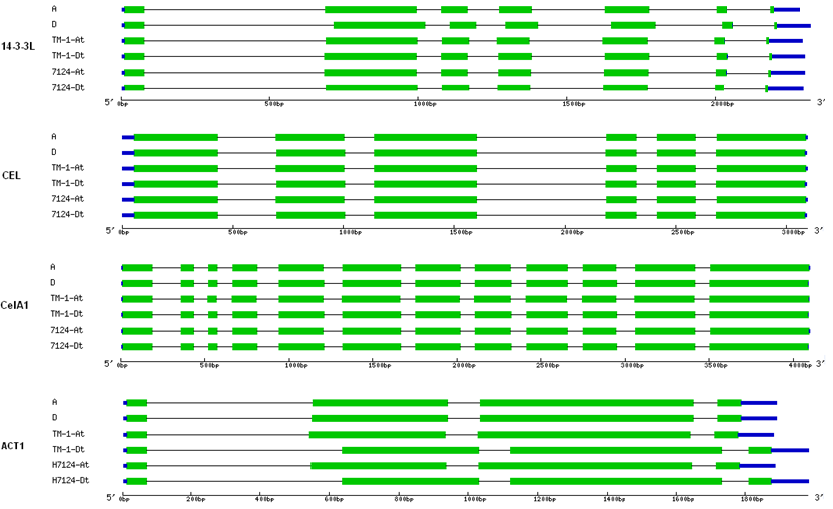

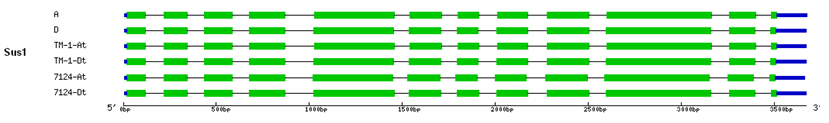

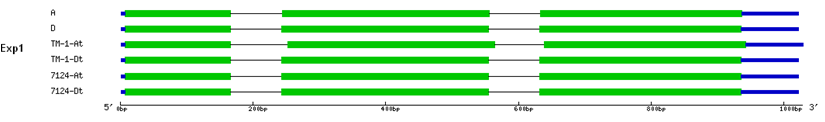

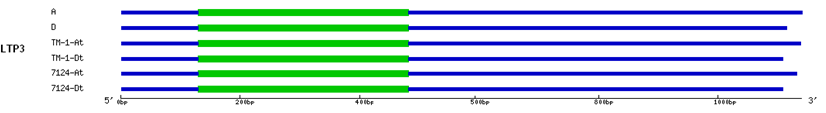


B


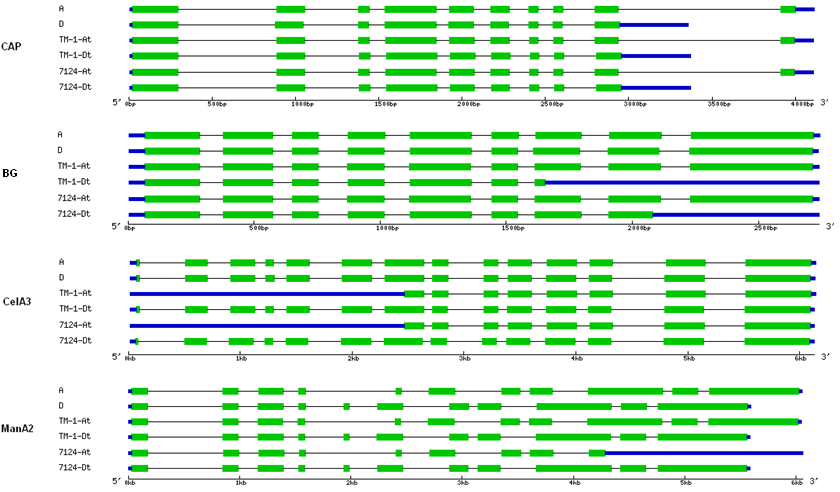

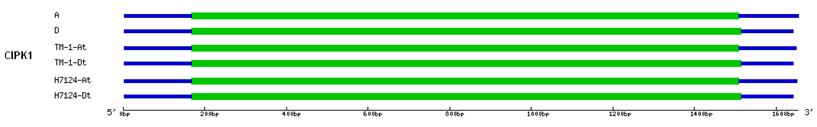

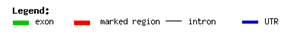


Figure S1. Structure analysis of fiber development-related genes in four cotton species. A: *G. herbaceum* L. var. *africanum*; D: *G. raimondii* Ulbr; TM-1-At: A subgenome of *G. hirsutum* L. acc. TM-1; TM-1-Dt: D subgenome of *G. hirsutum* L. acc. TM-1; 7124-At: A subgenome of *G. barbadense* L. cv. 7124; 7124-Dt: D subgenome of *G. barbadense* L. cv. 7124.

A. Orthologs having the same structures among four different cotton species.

B. Orthologs having different structures among four different cotton species.
