# Supplementary material for: Structure, expression differentiation and evolution of duplicated fiber developmental genes in Gossypium barbadense and G. hirsutum
Source: BMC Plant Biol. 2011 Feb 25;11:40. doi: 10.1186/1471-2229-11-40 (PMC3050799; doi:10.1186/1471-2229-11-40)
Supplement: Additional file 3 — Figure S2. The phylogenetic trees of 17 genes in four cotton species. Bootstrap values (%) based on 1000 replicates are indicated beside the nodes. A. Genes that evolved independently between the A- and D-subgenomes. B. Genes that evolved through different degrees of colonization between the A- and D-subgenome. [file 1471-2229-11-40-S3.DOC]

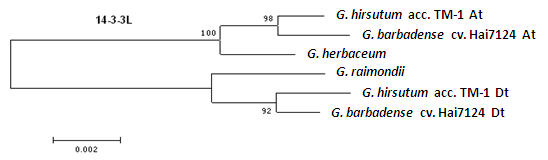

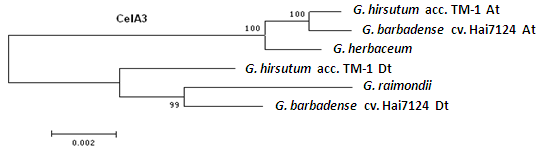

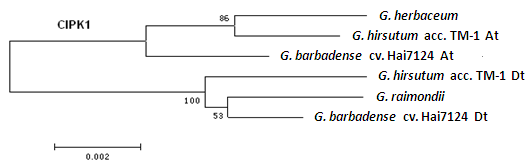

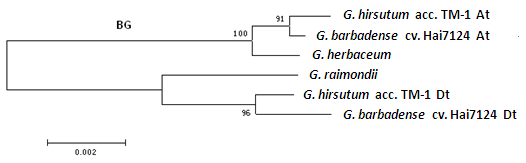

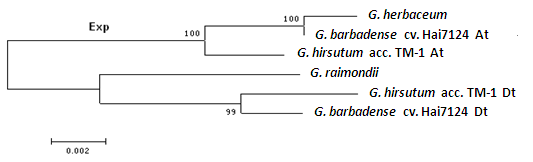


**A**


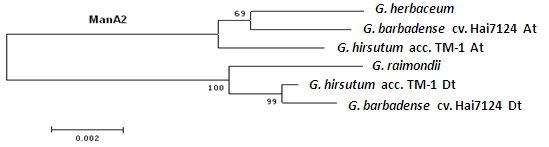

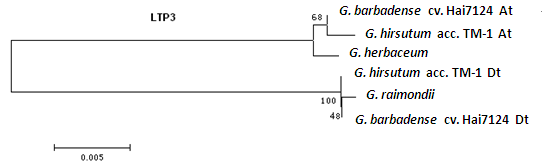

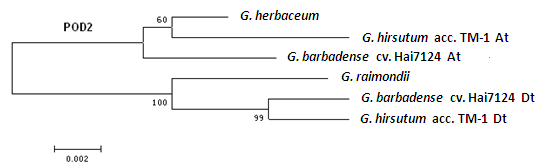

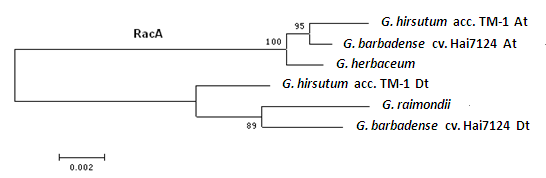

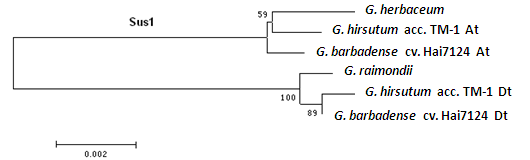

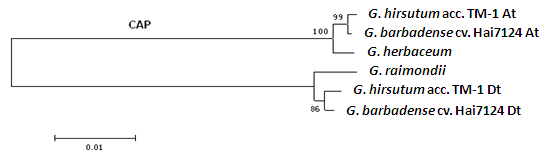

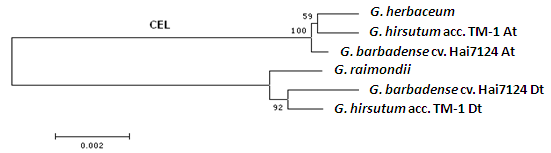

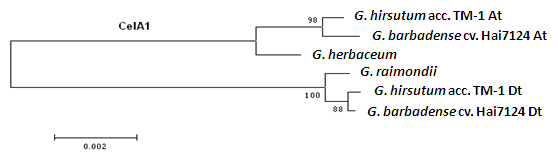

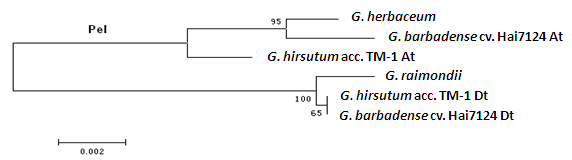

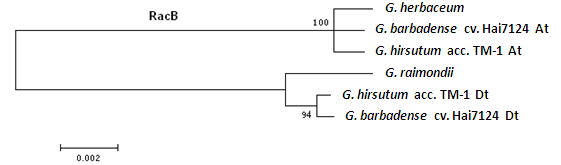


B


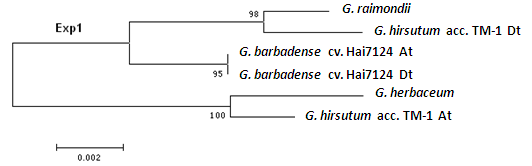

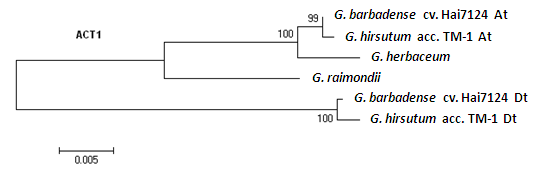


Figure S2. The phylogenetic trees of 17 genes in four cotton species. Bootstrap values (%) based on 1000 replicates are indicated beside the nodes.

A. Genes that evolved independently between the A- and D-subgenomes.

B. Genes that evolved through different degrees of colonization between the A- and D-subgenome.
