# Supplementary material for: Structure, expression differentiation and evolution of duplicated fiber developmental genes in Gossypium barbadense and G. hirsutum
Source: BMC Plant Biol. 2011 Feb 25;11:40. doi: 10.1186/1471-2229-11-40 (PMC3050799; doi:10.1186/1471-2229-11-40)
Supplement: Additional file 4 — Table S2. PCR primer pairs used for gene location. [file 1471-2229-11-40-S4.DOC]

Table S2. PCR primer pairs used for gene location.

| Gene | Subgenome | PCR primers(5’-3’) | |
| --- | --- | --- | --- |
|  |  | F | R |
| *CelA3* | At | GACAAAACAGACTGGGGAAG | TCACTAATCTGGGAGCACAA |
|  | Dt | ATTGATATTTGGACGACCTGTT | GCAAACCGGAGACAACTATG |
| *Sus1* | At | TTCCTCAGAGTCCATTGTCAC | CTGCAAAAAGAACAACTCAGC |
|  | Dt | - | - |
| *LTP3* | At | TGAGAGATTAGATCGTGGTGA | CTTCACAACATTAGGGCACA |
|  | Dt | - | - |
