# Supplementary material for: Structure, expression differentiation and evolution of duplicated fiber developmental genes in Gossypium barbadense and G. hirsutum
Source: BMC Plant Biol. 2011 Feb 25;11:40. doi: 10.1186/1471-2229-11-40 (PMC3050799; doi:10.1186/1471-2229-11-40)
Supplement: Additional file 5 — Table S3. SNP primer pairs used for gene location. [file 1471-2229-11-40-S5.DOC]

Table S3. SNP primer pairs used for gene location.

| Gene | Subgenome | SNP primers(5’-3’) | |
| --- | --- | --- | --- |
|  |  | F | R |
| *14-3-3L* | At | ATTTGTTATTTGGTAATCCAGGTGGGCATTT | GTCACTCTAGCAGCAAGCCCCTTTCC |
|  | Dt | GTTGAATTCCCTCCAAATCCTGCATC | CCAAGGAAAGTATTTCTGAAACTACCACAAAATGA |
| *CAP* | At | CGCACTTGGTGTGTGGGTACGACA | AGTTTTCAATTCAAATAGGTCTCATTTATGCTCCTG |
|  | Dt | TAAGTGAGTTCTGTCTAGTTGTTGGATTTTAAGGGG | TGGACAACACATCTGGCTGTCAGTTGTAC |
| *CEL* | At | - | - |
|  | Dt | CTGTCGGTGCTCCTGCATTGTCA | GACAGTCTTGACGATGAGGGTGATGAGTC |
| *CIPK1* | At | - | - |
|  | Dt | TGGGTTGTCGGCTTTGCCAGAT | ACCAAGGATCACGCATTATAGCTGGGAT |
| *BG* | At | CATGATCAATGACTTTCAGAAGTGG | GCAACATGCCTGGTAGCACCT |
|  | Dt | GAAGATTTAGCAGGCATGTCTCCTTGCAG | GTACATGTTACTCAGGGACCCTAAACTGGTTAAGAA |
| *POD2* | At | TGACGATGCCAAAACTCAGCTAGAACCTT | AATTACTTACCAACGAGAGCAACAAGGTCTTGA |
|  | Dt | CCTTGATTATAATCCCAGTCTACTAACTACGCG | ATGTTAGGATTAGAACGGAAATGGGACT |
| *RacA* | At | GGTCTAACTGAAGTCATGCATCTCGTAGAGCTA | AACTGGAAGTGTCACTAAATTGTGGACTTGATATTG |
|  | Dt | AAGCTTCAAACATAAAACTTAAAGCATAAAACTTCG | TTTTTGCTGCTAGAGTTTCCATTTATTCTTGTTTTT |
| *RacB* | At | CCCCTAAACTGACACCCCTAGAAAGCATG | TTTTTACTTTTTGCAATCTGTTCTCGCCAA |
|  | Dt | ACATAAATTTTCAGTTCAAAGATAATGAAGACGATA | TTGCCATTTCATGAATTTTTCTTATGTTTTCAACT |
| *Pel* | At | TGGGGTTAACGGGGTCATCAGCA | CCTCTTCCCCTGTTCAAGACCCTGAG |
|  | Dt | - | - |
| *ManA2* | At | CAGCTCACCACCACTCATCCCCTT | AAGTTGAAAAAGCTAACAACCAAGCAGAACCTAA |
|  | Dt | TCCAAACCATTAAGATGAGAACTCACATACTATCA | AAAAATTGTAGTTTTGTTGATATAACGCCATAATTG |
| *ACT1* | At | - | - |
|  | Dt | CGGCCTACATTACAGACAAAATTAGCTTAACCGTAT | AAGACACATTGCATTCCTAAGAGGAGACATAGATTT |
