# Supplementary material for: Structure, expression differentiation and evolution of duplicated fiber developmental genes in Gossypium barbadense and G. hirsutum
Source: BMC Plant Biol. 2011 Feb 25;11:40. doi: 10.1186/1471-2229-11-40 (PMC3050799; doi:10.1186/1471-2229-11-40)
Supplement: Additional file 6 — Figure S3. Q-PCR analysis for total expression of genes. Significant differences between TM-1 and Hai7124 at the same stages indicated by * P > 0.05, ** P > 0.01. Vertical bars represented standard deviation (STD). A. Genes expressed preferentially at fiber initiation and early elongation (0-8 DPA). B. Genes expressed preferentially at fiber elongation (3-17 DPA). C. Genes expressed preferentially at primary-secondary transition (17-23 DPA). D. Genes expressed both at fiber initiation and early elongation period (0-8DPA) and secondary cell wall thickening period (20-23DPA). E. Genes expressed coving the whole fiber developmental period. [file 1471-2229-11-40-S6.DOC]

A


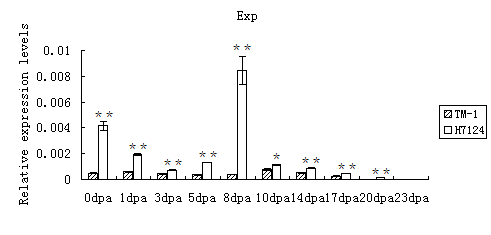

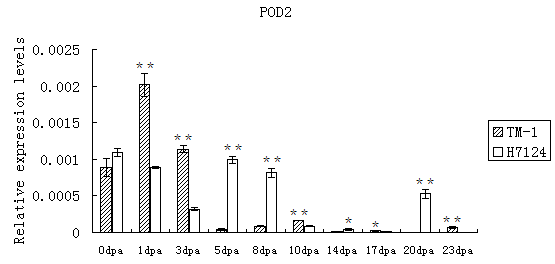


**POD2**


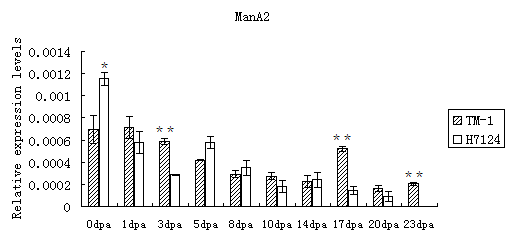


**ManA2**

B


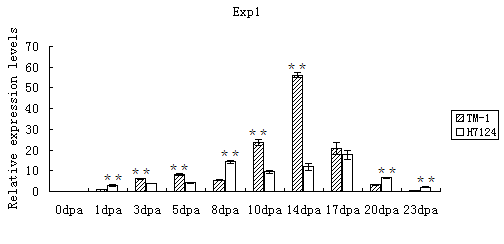


**Exp1**


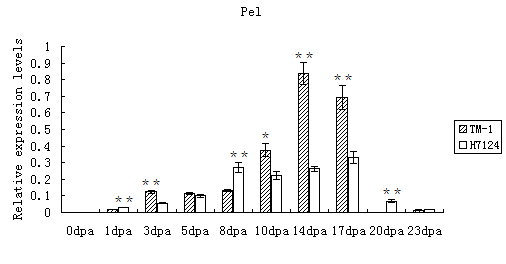


**Pel**


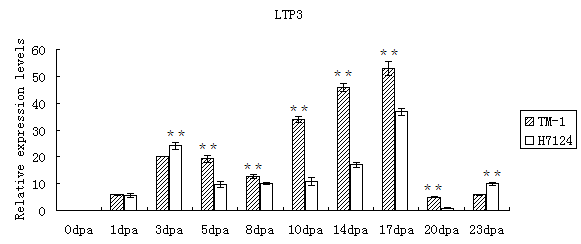


**LTP3**

**Exp**


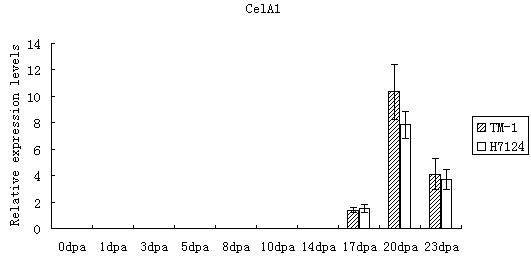


**CelA1**

C


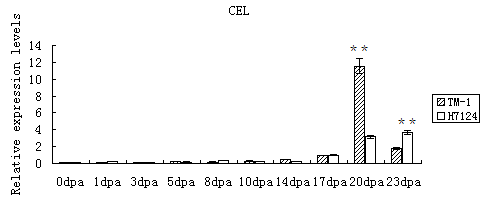


**CEL**


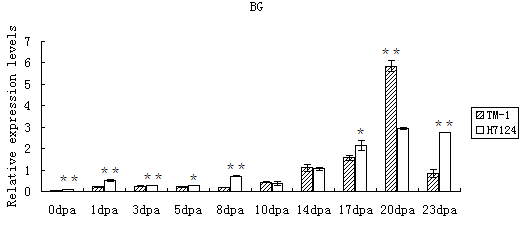


**BG**


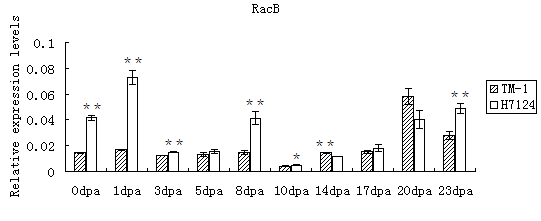


**RacB**

D


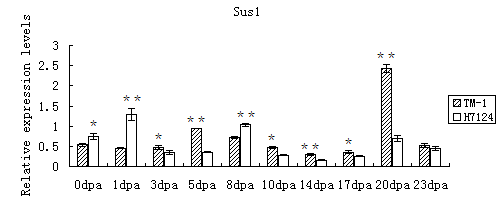


**Sus1**


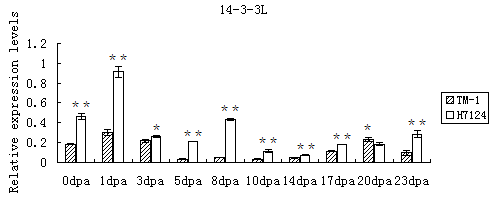


**14-3-3L**

E


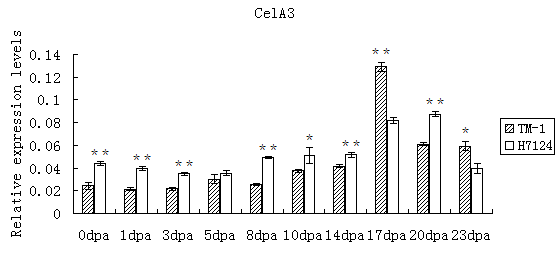


**CelA3**


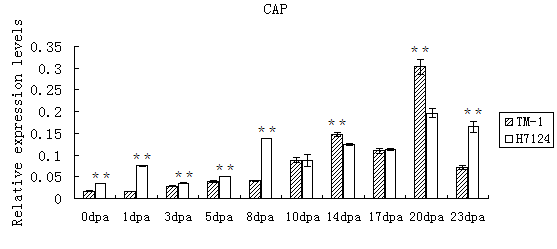


**CAP**


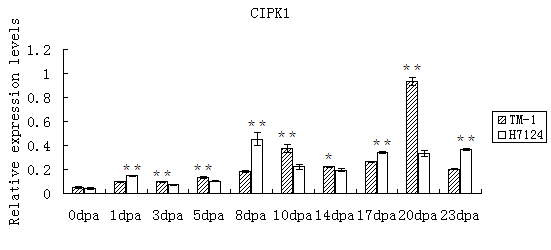


**CIPK1**


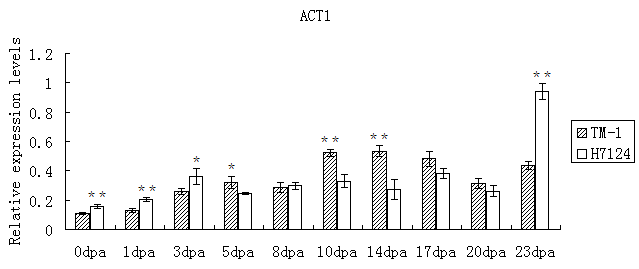


**ACT1**


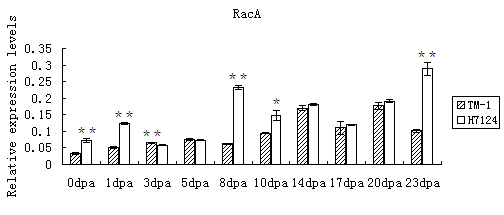


**RacA**

Figure S3. Q-PCR analysis for total expression of genes. Significant differences between TM-1 and Hai7124 at the same stages indicated by * P< 0.05, ** P< 0.01. Vertical bars represented standard deviation (STD).

A. Genes expressed preferentially at fiber initiation and early elongation (0-8 DPA).

B. Genes expressed preferentially at fiber elongation (3-17 DPA).

C. Genes expressed preferentially at primary-secondary transition (17-23 DPA).

D. Genes expressed both at fiber initiation and early elongation period (0-8DPA) and secondary cell wall thickening period (20-23DPA).

E. Genes expressed coving the whole fiber developmental period.
