# Supplementary material for: Structure, expression differentiation and evolution of duplicated fiber developmental genes in Gossypium barbadense and G. hirsutum
Source: BMC Plant Biol. 2011 Feb 25;11:40. doi: 10.1186/1471-2229-11-40 (PMC3050799; doi:10.1186/1471-2229-11-40)
Supplement: Additional file 7 — Table S4. Subgenomic-specific PCR primer pairs used for Q-PCR analysis. [file 1471-2229-11-40-S7.DOC]

**Table S4. Subgenomic-specific PCR primer pairs used for Q-PCR analysis**.

| Gene | Subgenome | SNP primers(5’-3’) | | Ampliﬁed product sizes (bp) in cDNA | Ampliﬁed product sizes (bp) in DNA |
| --- | --- | --- | --- | --- | --- |
|  |  | F | R |  |  |
| *CAP* | At | ATTGATCTCACTTGACTTTGCAGA | AGAACCAAATTGGAAGAAAGAACTTA | 320 | 720 |
|  | Dt | ATTGATCTCACTTGACTTTGCAGT |  | 320 | 720 |
| *CEL* | At | CAAAGCATGTTCACCATAGAGAG | TGGTGTAGGAAACATTGGTGG | 305 | 305 |
|  | Dt | TTGTGGGAGCCATGGTAGTT | TTTCATGGTTTCCAAGGTGC | 229 | 229 |
| *CIPK1* | At | AAACCCATATTATGCATTATTGTAGC | GTTTGTGACCGCTGAGGTGT | 345 | 345 |
|  | Dt | AAACCCATATTATGCATTATTGTTCT |  | 345 | 345 |
| *BG* | At | AACACAGTTGATTCAAGCACGA | CATAGTCACCAAACAGAACCTCA | 328 | 328 |
|  | Dt | AGGTATAGTTAGCATGAGGTGGAAAC | GTAGGAGATGGTGGAACTACCAAG | 279 | 279 |
| *Exp1* | At | TTCAACATGGTGTTGATGACG | TAATATTGTCTTAAAACTGGCCTCCT | 259 | 259 |
|  | Dt | TACTTCAACATGGTGTTGATACCC | TAATATTGTCTTAAAACTGGCCTCCT | 262 | 262 |
| *POD2* | At | GGCGCATTGATTTGGAGACT | AGTTAATGGCAGAGCAAATTCTTC | 270 | 270 |
|  | Dt | AACTCACAATCAATTACATCCACAGT | CATGACAGTCATTGCAAGGAAG | 145 | 145 |
| *Exp* | At | TCAGAACTGGCAGTCAAATGAA | GAGGGGTTGCAAAATCTATCAA | 169 | 169 |
|  | Dt | TCAGAACTGGCAGTCAAATGAG |  | 169 | 169 |
| *RacB* | At | CTCCGAGATGATCAACAATTCTAA | GTTCCATCTTTTTCAGACTTGAAGA | 262 | 453 |
|  | Dt | CCGAGATGATCAACAATTCTCG |  | 260 | 451 |
| *Pel* | At | GATTCAGCAAAGAGGTGACCATA | CATCAACAACGTGAACCTTTCTT | 238 | 238 |
|  | Dt | GGGAATAGATTTACTGCACCCG |  | 266 | 266 |
| *ManA2* | At | ATGTGAAGGGACACGGAGTG | CTCTACCATCAACATCACCAGTAATC | 339 | 745 |
|  | Dt | CTTTTAAGGAATCACCCTAGTCTAGC | TTCCTACAGAACCAACCTCAGG | 343 | 343 |
| *CelA1* | At | TTTCCTTTCCATTATCGTGAGC | TGACCCAGAAGGAAAAGAACAC | 368 | 368 |
|  | Dt | TTCCTTTCCATTATCGTGGCT |  | 367 | 367 |
| *CelA3* | At | CATGAGAAGAGGAATAGCAGTGAGT | TTCTTACTGGATTCAAAATGCATG | 263 | 263 |
|  | Dt | ATGAGAAGAGGAATAGCAGTGAGC |  | 262 | 262 |
| *Sus1* | At | TGCCAACATTCGCAACTTGT | TCTTATCCCAGTGAGATGGATCTT | 156 | 156 |
|  | Dt | AATCTAAAGATTTGGAAGAGAAGGAC | TCTTATCCCAGTGAGATGGATCTT | 375 | 375 |
| *14-3-3L* | At | GGTCGAAGATGAACTCTCAACG | ATAGCCTCATCAAATGCTTGTTTAG | 345 | 780 |
|  | Dt | AGGGTTGAAGATGAGCTCTCAATA |  | 347 | 782 |
| *RacA* | At | GCTATACAAGTAACAAGTTCCCAACA | AGGATGATCAGCCAGATAATGTTT | 326 | 1836 |
|  | Dt | CAAGTAACAAGTTCCCGCCC | TCTGTTTGCGGAGCTCCTCGC | 369 | 369 |
| *LTP3* | At | GCAAGTATTTTCCTTACACG | TACACAACGCATAACTCTGG | 576 | 576 |
|  | Dt | GCAAGCATTTTCCTTACAAG | TACACAACCCACAACTCTGG | 586 | 586 |
| *ACT1* | At | CCATGTTCCCCGGTATTGAA | AATGTAGATCACCATCAAAAGCAAT | 329 | 329 |
|  | Dt | CTATGTTCCCTGGTATCGCG |  | 329 | 329 |
| *EF1α* |  | AGACCACCAAGTACTACTGCAC | CCACCAATCTTGTACACATCC | 495 |  |
