# Supplementary material for: Structure, expression differentiation and evolution of duplicated fiber developmental genes in Gossypium barbadense and G. hirsutum
Source: BMC Plant Biol. 2011 Feb 25;11:40. doi: 10.1186/1471-2229-11-40 (PMC3050799; doi:10.1186/1471-2229-11-40)
Supplement: Additional file 8 — The information of the Q-PCR analysis based on the MIQE checklist. [file 1471-2229-11-40-S8.DOC]

| **EXPERIMENTAL DESIGN** |
| --- |
| Definition of experimental and control groups |
| Number within each group |
| Assay carried out by core lab or investigator's lab? |
| Acknowledgement of authors' contributions |
| **SAMPLE** |
| Description |
| Volume/mass of sample processed |
| Microdissection or macrodissection |
| Processing procedure |
| If frozen - how and how quickly? |
| If fixed - with what, how quickly? |
| Sample storage conditions and duration (especially for FFPE samples) |

**The information of the Q-PCR analysis based on the MIQE checklist**

Experimental design is provided in the material and method section. RNA was isolated from two different species with three biological replicates at 10 given developmental periods. Assays were carried out in the investigator’s lab.

In general, samples at 0, 1, 3, 5 DPA were ovule and fiber mixture, and that at 8, 10, 14, 17, 20 and 23 DPA were only fiber tissues. All samples were collected and frozen immediately in liquid nitrogen. Further, about 0.5-1g of samples was used for RNA extraction.

| **NUCLEIC ACID EXTRACTION** |
| --- |
| Procedure and/or instrumentation |
| Name of kit and details of any modifications |
| Source of additional reagents used |
| Details of DNase or RNAse treatment |
| Contamination assessment (DNA or RNA) |
| Nucleic acid quantification |
| Instrument and method |
| Purity (A260/A280) |
| Yield |
| RNA integrity method/instrument |
| RIN/RQI or Cq of 3' and 5' transcripts |
| Electrophoresis traces |
| Inhibition testing (Cq dilutions, spike or other) |

The RNA isolation procedure is described in Jiang and Zhang (2003), no kit was used to isolate RNA. To remove any remaining DNA traces, 50µg RNA was treated with 10U of Dnase I (RNase free, TaKaRa, Code No. D2215) and 40U Ribonuclease Inhibitor (TaKaRa, Code No. D2313) in a 100µl volume. All following procedures were performed according to the manufacturer’s instructions. Contamination was assessed by a direct use of treated RNA in the qPCR reaction; if the control gene (e.g. *EF1α* and *his3*) is detectable by directly amplifying treated RNA, there existed a DNA contamination in treated RNA. Additionally, the test gene used for qPCR (e.g. *CAP*) is flanking an intron and since a melting curve is performed as standard, a contamination would be visible as additional peak. The precipitated RNA was resuspended in DEPC treated water. A 1:50 dilution was measured with the Eppendorf BioPhotometer plus. The A260/280 ratio is generally between 1.9 and 2.0, exact values for each RNA sample can be provided upon request. The average yield is about 20-100μg of RNA each 1g fresh tissues (because different tissues have different RNA yield, young tissues have the more extraction rate, e.g. young ovules at 0DPA).

RNA integrity was detected by electrophoresis using 1% agarose gel. 28S and 18S RNA for each sample in electrophoresis was clear and the brightness of 28S was more than two fold of 18S. As the primers of control gene is located 5’ transcript region, PCR was performed before assays. At the same time, position of the threshold is standardized and the Cq values difference for the control gene *EF1α* was within 1 cycle for all cDNAs used. Inhibition testing was not performed.

| **REVERSE TRANSCRIPTION** |
| --- |
| Complete reaction conditions |
| Amount of RNA and reaction volume |
| Priming oligonucleotide (if using GSP) and concentration |
| Reverse transcriptase and concentration |
| Temperature and time |
| Manufacturer of reagents and catalogue numbers |
| Cqs with and without RT |
| Storage conditions of cDNA |

AMV Reverse Transcriptase XL from TaKaRa (Code No. D2620, 5U/µl) was used for the generation of first strand cDNA in a 25µl reaction volume. 1µg of RNA (5~10µl), 1µl of oligo(dT)_18_ (500µg/ml) and 1µl dNTP mix (Invitrogen, Catalog No. 18427) were incubated at 70℃ for 10 min and quick chilled on ice. All other steps were performed according to manufacturer’s instructions except that the incubation time at 42℃ was increased from 60 to 90 min. For most cDNAs, the internal control *EF1α* and *his3* amplicon was used for the estimation of contamination. Each analysis contained a negative control (without cDNA template) to evaluate the overall specificity. cDNA was stored in low adhesion tubes at -20℃.

| **qPCR TARGET INFORMATION** |
| --- |
| If multiplex, efficiency and LOD of each assay. |
| Sequence accession number |
| Location of amplicon |
| Amplicon length |
| *In silico* specificity screen (BLAST, etc) |
| Pseudogenes, retropseudogenes or other homologs? |
| Sequence alignment |
| Secondary structure analysis of amplicon |
| Location of each primer by exon or intron (if applicable) |
| What splice variants are targeted? |

#### Multiplex qPCR was not performed. Sequence accession numbers are included in Table1 in the MS. Development of homeolog-specific PCR primer pairs is provided in the material and method section. Amplicon length is included in TableS4 under qPCR validation. In silico screen were performed with NCBI Blast. Primers were designed in exons or UTR regions close to the 3’ end of the gene. No splice variants were targeted.

| **qPCR OLIGONUCLEOTIDES** |
| --- |
| Primer sequences |
| RTPrimerDB Identification Number |
| Probe sequences |
| Location and identity of any modifications |
| Manufacturer of oligonucleotides |
| Purification method |

Primer sequences are included in the manuscript as supplemental file 4. No modifications were used. Primers were synthesized by Genscript Biotechnology Co., Ltd (Nanjing, China) and are salt-free.

| **qPCR PROTOCOL** |
| --- |
| Complete reaction conditions |
| Reaction volume and amount of cDNA/DNA |
| Primer, (probe), Mg++ and dNTP concentrations |
| Polymerase identity and concentration |
| Buffer/kit identity and manufacturer |
| Exact chemical constitution of the buffer |
| Additives (SYBR Green I, DMSO, etc.) |
| Manufacturer of plates/tubes and catalog number |
| Complete thermocycling parameters |
| Reaction setup (manual/robotic) |
| Manufacturer of qPCR instrument |

Each qPCR reaction had a 20µl reaction volume containing:

cDNA corresponding to 20ng input RNA

0.4µM of each forward and reverse primer

10µl FastStart Universal SYBR Green Master (ROX, 2×SYBR)（Roche）

( Cat.No. 04913850001）

Add ddH_2_O to 20µl

Tubes and lids were purchased from ABI (Code No. 4314320, Code No. N8010580 and 4323032)

Cycling parameters were:

95℃ for 10 min,

94℃ for 10 sec

60-65℃ for 20 sec

72℃ for 25 sec

Plate read

80℃ for 1 sec

Plate read

Cycle 42 times

Melting curve from 65°C to 95°C, read every 0.2°C, hold 1 sec

Reactions were set up manually in a designated room using designated equipment. qPCRs were performed with the [7500 Real-Time PCR System](http://marketing.appliedbiosystems.com/mk/submit/MOUSESNPBROWSER_RD?_JS=T&rd=q) from Applied Biosystems.

| **qPCR VALIDATION** |
| --- |
| Evidence of optimisation (from gradients) |
| Specificity (gel, sequence, melt, or digest) |
| For SYBR Green I, Cq of the NTC |
| Standard curves with slope and y-intercept |
| PCR efficiency calculated from slope |
| Confidence interval for PCR efficiency or standard error |
| r2 of standard curve |
| Linear dynamic range |
| Cq variation at lower limit |
| Confidence intervals throughout range |
| Evidence for limit of detection |
| If multiplex, efficiency and LOD of each assay. |

The specificity of the amplification products have been confirmed by size estimations on a 2% agarose gel, by sequencing of the products and by analyzing their melting curves. Without a template, no Cq could be determined since it never passed the threshold line. Reference genes were used for efficiency determination of each template. Calculated PCR efficiency of each template was over 90%. For example, standard curves with slope and y-intercept for reference genes at 0DPA in TM-1 are following:

| **Reference genes** | **length (bp)** | **slope** | **y-intercept** | **% efficiency** | **r^2^** |
| --- | --- | --- | --- | --- | --- |
| *EF1α* | 495 | -3.2322 | 16.246 | 103.9% | 0.9893 |
| *his3* | 127 | -3.5423 | 16.343 | 91.6% | 0.9995 |

| **DATA ANALYSIS** |
| --- |
| qPCR analysis program (source, version) |
| Cq method determination |
| Outlier identification and disposition |
| Results of NTCs |
| Justification of number and choice of reference genes |
| Description of normalisation method |
| Number and concordance of biological replicates |
| Number and stage (RT or qPCR) of technical replicates |
| Repeatability (intra-assay variation) |
| Reproducibility (inter-assay variation, %CV) |
| Power analysis |
| Statistical methods for result significance |
| Software (source, version) |
| Cq or raw data submission using RDML |

qPCR analysis program (source, version): ABI, SDS Software

Cq’s were determined by setting the threshold automatic (10×standard deviation of baseline) using a log scale.

No data have been excluded from the calculations.

Results of NTCs: no amplification products present thus no Cqs

Justification of number and choice of reference genes: cDNAs had previously been tested with

another reference gene (*his3*) with the same results

Description of normalisation method: endogenous reference gene

Number and concordance of biological replicates: 3

Number and stage (RT or qPCR) of technical replicates: 3 at qPCR level, 2 for RT analysis

Repeatability (intra-assay variation): was below one Cq

Statistical methods: ANOVA and least signification difference (LSD)

Software (source, version) stst 1.00
